# Question classification based on Bloom’s taxonomy cognitive domain using modified TF-IDF and word2vec

**DOI:** 10.1371/journal.pone.0230442

**Published:** 2020-03-19

**Authors:** Manal Mohammed, Nazlia Omar

**Affiliations:** 1 CAIT, Faculty of Information Science and Technology, Universiti Kebangsaan Malaysia, Bangi, Selangor, Malaysia; 2 Management Information Systems department, Faculty of Administrative Science, Hadhramout University, AL-Mukalla, Yemen; Newcastle University, UNITED KINGDOM

## Abstract

The assessment of examination questions is crucial in educational institutes since examination is one of the most common methods to evaluate students’ achievement in specific course. Therefore, there is a crucial need to construct a balanced and high-quality exam, which satisfies different cognitive levels. Thus, many lecturers rely on Bloom’s taxonomy cognitive domain, which is a popular framework developed for the purpose of assessing students’ intellectual abilities and skills. Several works have been proposed to automatically handle the classification of questions in accordance with Bloom’s taxonomy. Most of these works classify questions according to specific domain. As a result, there is a lack of technique of classifying questions that belong to the multi-domain areas. The aim of this paper is to present a classification model to classify exam questions based on Bloom’s taxonomy that belong to several areas. This study proposes a method for classifying questions automatically, by extracting two features, TFPOS-IDF and word2vec. The purpose of the first feature was to calculate the term frequency-inverse document frequency based on part of speech, in order to assign a suitable weight for essential words in the question. The second feature, pre-trained word2vec, was used to boost the classification process. Then, the combination of these features was fed into three different classifiers; K-Nearest Neighbour, Logistic Regression, and Support Vector Machine, in order to classify the questions. The experiments used two datasets. The first dataset contained 141 questions, while the other dataset contained 600 questions. The classification result for the first dataset achieved an average of 71.1%, 82.3% and 83.7% weighted F1-measure respectively. The classification result for the second dataset achieved an average of 85.4%, 89.4% and 89.7% weighted F1-measure respectively. The finding from this study showed that the proposed method is significant in classifying questions from multiple domains based on Bloom’s taxonomy.

## 1. Introduction

**Fig 1:**
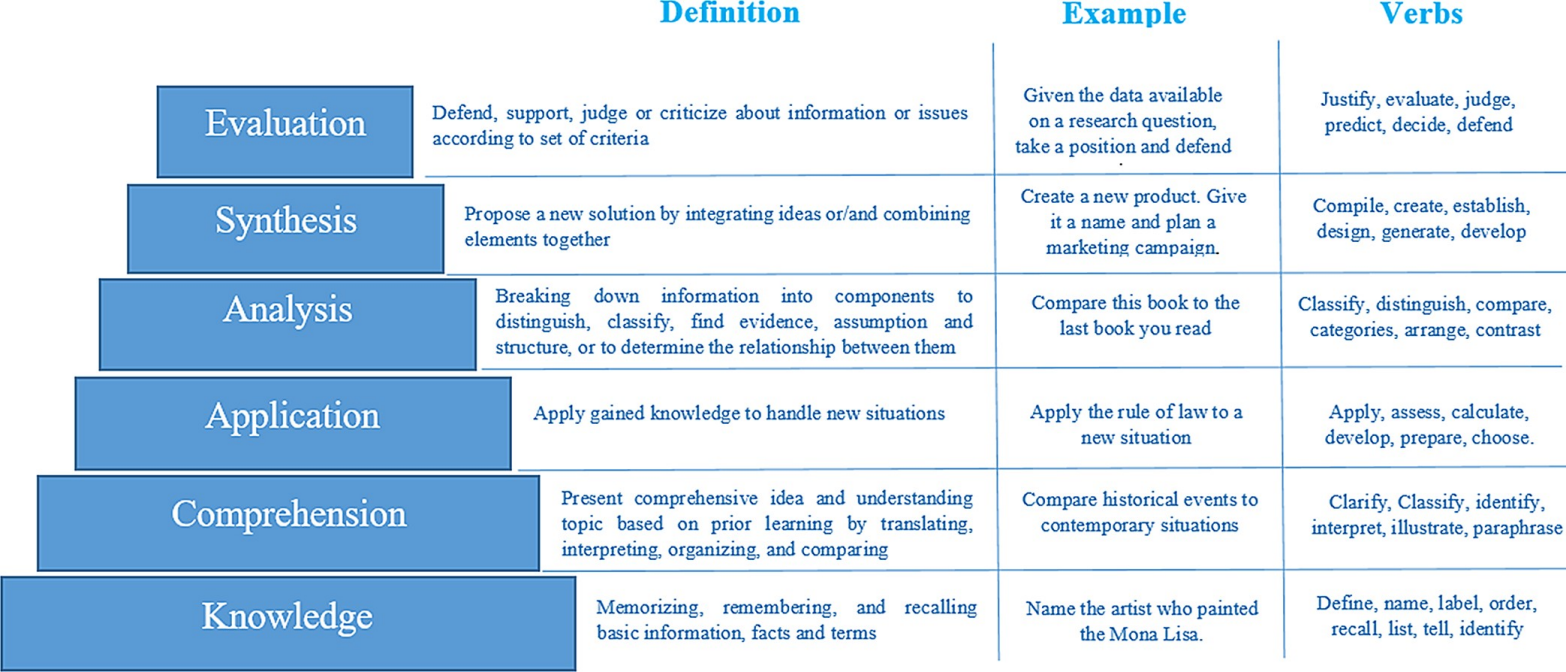
Bloom’s taxonomy cognitive domain levels.

Examination assessment plays a fundamental role in evaluating how students’ proficiency in the course content [1]. Writing high quality and balanced exam questions that satisfy different levels of cognition is not an easy task [2]. Constructing the examination in a comprehensive manner must also take into consideration the difficulty levels, that match the standard objectives and outcomes of the course in a standard way such as Bloom’s taxonomy [1,3]. Bloom’s taxonomy involves three domains, i.e. cognitive, affective, and psychomotor domains. The cognitive domain, covers different thinking skills starting from the most straightforward to the most complex one [4,5] as shown in Fig 1. The key to writing learning outcomes by Bloom’s taxonomy is verbs [4].

Recently, researchers [5–9] have shown an increased interest in automating evaluating examination based on Bloom’s taxonomy cognitive domain. Various techniques have been used along with different features such as lexical features, and syntactic features, while few of them used semantic features. On the other hand, most previous works handled classifying questions from a specific domain, where there is a lack of techniques on classifying questions based on Bloom’s taxonomy over the multi-domain area [10,11]. Therefore, this study aims to build a question classification model based on Bloom’s taxonomy cognitive domain. Hence, considerable room for further improvement still remains, particularly in open domain area.

In general, questions classification is unlike documents classification, since the questions are written in short forms. Documents classification aids users to get and extract useful information easily due to the extensive available information. Whereas, short text suffers from the lack of gained information and sparsity [12,13]. Thus, it is not suitable to use the pure statistical method in order to perform question classification such as N-gram and TF-IDF, since these techniques need a massive amount of data in order to get high accuracy [5]. In addition, removing stop-words during the text classification pre-processing stage of the document is a common step to reduce insignificant words. Nevertheless, some of these stop-words such as *what*, *when*, *where*, and *how* are valuable in the process of question classification [14].

Another main issue in classifying questions based on Bloom’s cognitive is assigning a suitable weight for keywords that determine the level of the question, especially for the words that might appear in more than one taxonomy, such as the word *‘define’* that belongs to the *Knowledge* and *Comprehension* levels. To handle this issue, [15,16] proposed assigning weight to the words from the perspective of experts. Using this method may lead to inconsistencies due to the variety of background knowledge of each expert. This resulted in the poor performance of the classification process.

Furthermore, there are still other methods to classifying questions based on Bloom’s cognitive level which might not have been investigated yet such as using word embedding, e.g., word2vec which have shown good results in sentiment analysis and question classification in the question answering system [17–19]. Thus, it might be useful to gain benefit from the use of word embedding with a combination of the modified feature TF-IDF, to enhance the classification process.

## 2. Related work

The issue of classifying exam questions based on Bloom’s taxonomy has received a considerable critical attention in recent years. In order to handle this task, researchers use different techniques and features. For example, [15] used a pre-defined list of keywords on their online test system, in which the verbs on the questions checked against the keyword list. Their dataset consists of 228 questions written in English. This method can only classify questions that belong to the knowledge level. A rule-based approach with natural language processing techniques was used by [16] to return significant words in a question, then assign weights to these words which are defined previously by experts. Their dataset consists of 100 computer programming questions written in English where the dataset is split into 70% training set and 30% test set. Similarly, [2] used a rule-based approach with N-gram, to enhance the classification result and produce 86% average F1. The dataset that is used in this work consists of 135 questions in computer programming subject written in English language which is split into 100 questions as a training set and 35 questions as a testing set. Likewise, [8] used rules to classify questions from three subjects; Object-oriented programming, software engineering and algorithm and data structure. The dataset contains 53 questions written in English. This work [8] used WordNet with cosine similarity to define question pattern. The accuracy of the outcome from the classification process reached 71%.

However, other researchers [5,6,20–24] used machine learning techniques to classify Bloom’s taxonomy into cognitive levels. A neural network is used by [22] with different feature sets: whole feature, document frequency, and category frequency-document frequency to classify questions by training the model with a scaled conjugate gradient learning algorithm. The result shows that document frequency reduction is more effective with regard to the classification and coverage time compared to other feature reductions. Their dataset consists of 274 questions, divided into 192 training set and 82 testing set.

TF-IDF feature is extracted extensively in many works, [23] performed pre-processing for the dataset which contains 272 questions collected from different websites. Then TF-IDF was calculated and fed into Linear SVM, which produced satisfactory results in term of accuracy and precision, but not in F-measure and recall. This work was extended in [20] by increasing the size of the dataset to 600 questions and testing the effect of classification with and without stop words using the same TF-IDF feature. As a result, removing stop words does not make a big impact on the result. Similarly, [6] used the same dataset prepared by [20], but with enhanced TF-IDF which is multiplied with impact factor. Then the classification process handled by three classifiers NB, KNN, and SVM where SVM performance superior other classifiers. In addition, an ensemble technique is used by [5,24] to classify questions into Bloom’s taxonomy. [5] applied voting algorithm on a combination of three classifiers i.e. SVM, KNN and NB with chi-square, mutual information & odd ratio on questions from programming subject. [24] used ensemble to handle keywords overlapping issue by combining four classifiers namely rule-based, SVM, KNN and NB using majority voting algorithm and WordNet similarity. The dataset that is used in this work contains 100 programming questions which divided into 60 questions training set and 40 questions test set. The results show that the ensemble returns the best performance compared to each individual classifier by achieving 95% F-measure.

One of the most popular weighting methods is TF-IDF, which stands for Term Frequency- Inverse Document Frequency. It assigns scores to the importance of a word in a document, based on the lexical and morphological properties of the text. It is extensively used in many studies [25–29], and in question classification [3,6,20,23,30–32] However, some researchers used TF-IDF as it is, while others proposed some enhancement to the way TF-IDF is calculated in order to improve it. This is because classical TF-IDF does not capture some useful information such as the impact of word distribution between various classes [6].

A Massive Open Online Courses (MOOC) search engine proposed by [26], used a modified TF-IDF, that assigned the important words in the query to specific weights according to their parts of speech. This study changed the way of calculating the term ‘frequency’ by assigning a higher weight for the most important word, which is a noun or verb. Then in the second priority, the adjective and adverb are assigned to the weight that is less than a noun and verb but higher than other parts of speech which are assigned to the lowest weight. This method produces a significant result. Other researchers [33–35] also use the syntactic feature of the parts of speech to assign a suitable weight to the terms. Another example of improving the way of calculating the TF-IDF is proposed by [36] who introduced an impact factor that is multiplied by the traditional TF-IDF. The purpose of the impact factor equation is to calculate the weights of the class distribution. This enhancement performs better than the classical method. Similarly, [6] used the impact factor to enhance the result of TF-IDF to classify question based on Bloom’s taxonomy.

A comprehensive review in question classification by [37] mentioned that most of the works in question classification used semantic and syntactic features rather than pure statistical features such as bag-of-word and n-gram. Moreover, [37] stated that semantic features have been significantly used in question classification in question answering system and information retrieval and produced considerable accuracy, while there is a lack of extracting semantic features in educational environments.

On the other hand, many Natural Language Processing (NLP) studies with deep learning models have included learning word vector representation. The word vectors are represented in a dense form known as word embedding, in which the words that are semantically and syntactically related are close to each other in the embedding space [13,38–40]. Word embedding has been used efficiently in many NLP tasks [41].

Word2vec is one of the common word embeddings, where vectors representation is learnt via neural-network language model. It was developed based on Google by [38]. It required extensive training text as input and established vocabulary, then the model learned word vectors representation in such a way that the words that shared the same context are close to each other in the vector space. These word vectors can be used as features in various NLP tasks. As [17] mentioned, the pre-trained vectors can be considered as universal feature extractors. Many NLP tasks had benefited by word2vec, e.g., sentiment analysis, machine translation, and paraphrase detection [38]. [17] proposed a model that classified questions in question answering system, and sentiment analysis using Convolutional Neural Network (CNN) that was trained on the top of pre-trained word2vec, which produced excellent results. Similarly, [18] used CNN for Arabic sentiment analysis with Arabic pre-trained word2vec, which produced a significant result and outperformed previously existed methods.

The work [42] proposed a method that classified texts via SVM classifier, with the use of a semantic feature based on word2vec weighted by TF-IDF. In this study, several experiments compared if TF-IDF was better than combining it with word2vec as well as whether with or without stop words. Three steps were performed to produce the vector representation of word2vec with TF-IDF. The first step was summing the word2vec vectors to produce a single vector. The second step was the summation of multiplication of word2vec and TF-IDF. The third step was to concatenate the outcome of first and second step. For example, if the size of the vector of the first step was 200, and the vector size of the second step was 2000, then after appending them together the final representation of the vector would be 2200 which represented a document. The result showed that the best performance was achieved by word2vec without stop words being combined with TF-IDF without stop words.

Another example of using a combination of word2vec and TF-IDF was [36]. This work used three classifiers; SVM, KNN and Radial Basis Function (RBF) network, in order to classify Chinese documents. They performed enhancement to the TF-IDF by multiplying with impact factor, the improved feature called ITF-IDF. Then the ITF-IDF was combined with word2vec by applying the following equation:

$$\alpha_{d}=\frac{1}{J}\sum_{t\in d}^{}\text{word}2\text{vec}\left(\text{t},\text{d}\right).ITF-IDF\left(t,d\right)$$ (1)

where *t* is a term in document *d*, and *J* is the number of words in document *d*. For example, if the size of word2vec vectors is 200, then the size of the vector representation of the document can be obtained by applying Eq (1) is 200. The result of this study showed that the combination of both features outperformed the using of each feature individually with all classifiers.

The work of [32] classified questions based on a Persian question answering system. This study proposed three feature extraction methods; one of them was word2vec with weighted TF-IDF. The TF-IDF was raised to the power of TF-IDF factor and then multiplied by word2vec. The study stated that tuning the value of TF-IDF factor helped enhanced the accuracy significantly. SVM and Neural Network used as classification algorithms, in which they produced results that showed the effectiveness of the vector representation.

## 3. Method

**Fig 2:**
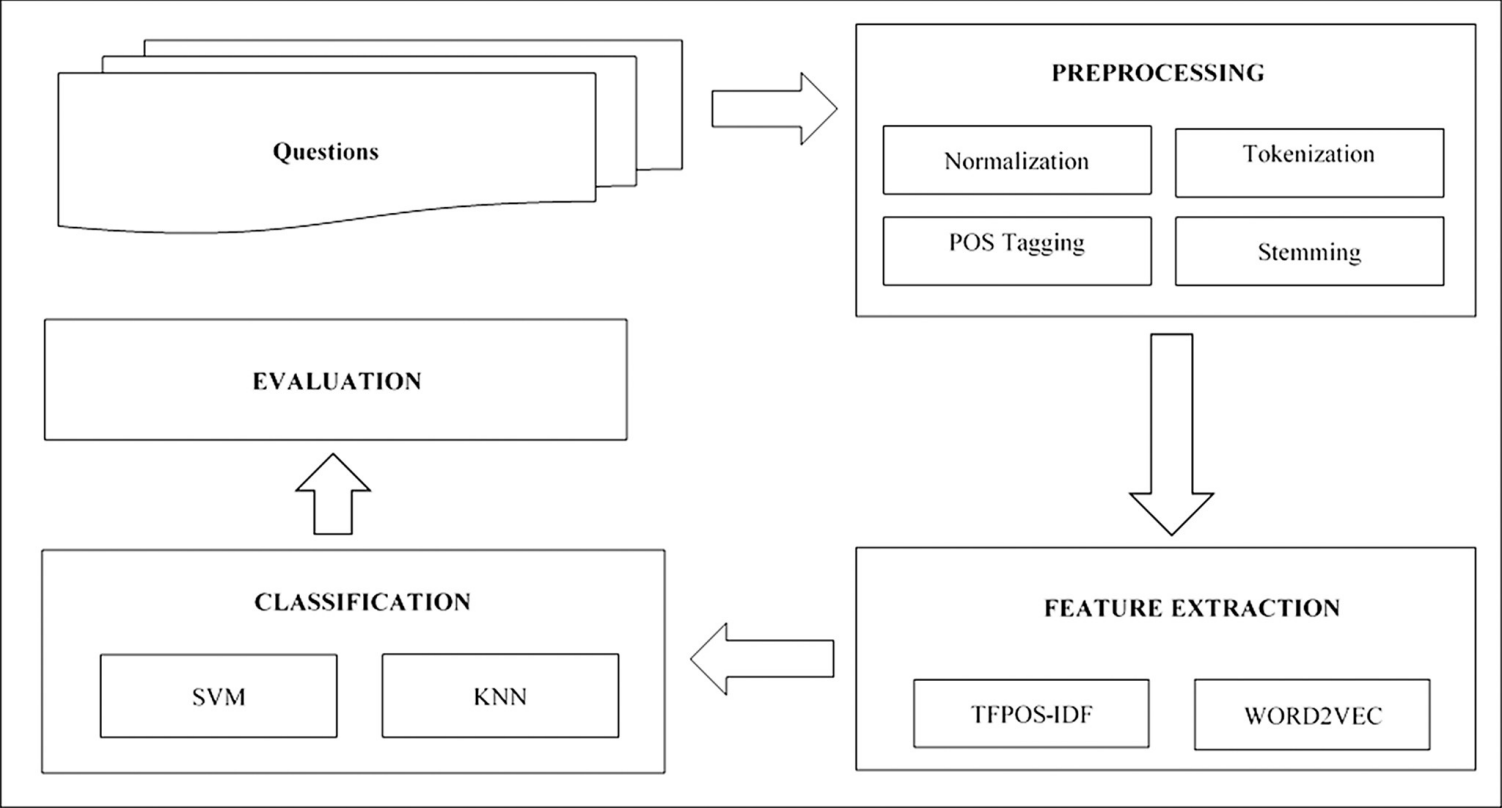
Proposed model.

The process of the proposed model is shown in Fig 2, and it involves five stages. The details of these stages will be discussed in detail in this section.

### 3.1 Question collection

Since the aim of this study was to build a model to classify questions based on Bloom’s Taxonomy from different domains, two open domain datasets had been used to ensure the stability of the proposed method. The first dataset were collected from several websites, books, and previous work [2]; the total number of questions in this dataset was 141 open-ended questions. The second dataset was introduced by Yahya et al. (2012), and it consisted of 600 open-ended questions. Both datasets were labeled and annotated into six classes. Table 1 shows the distribution of questions at each level in both datasets. Table 2 shows a sample of questions from both datasets.

**Table 1:** The number of questions in each dataset.

Cognitive Level	Collected Dataset	Yahya et al. (2012)
Knowledge Level	26	100
Comprehension Level	23	100
Application Level	15	100
Analysis Level	23	100
Synthesis Level	30	100
Evaluation Level	24	100
**Total**	**141**	**600**

**Table 2:** Sample of questions from each dataset.

	Collected dataset		Yahya et al. (2012) dataset	
Level	Question	Area	Question	Area
**KNOWLEDGE**	Memorize and recall the periodic table	Chemistry	Label the parts of the microscope shown on the right	Biology
	Name three 19th-century women English authors.	Literature	List reserved words in C programming.	Computer programming
**COMPREHENSION**	Explain how the heart is like a pump.	Biology	Retell the story in your words.	Literature
	Draw a diagram explaining how air pressure affects the weather.	Physics	Determine the next number in a sequence	Math
**APPLICATION**	Design or sketch a marketing strategy for your product using a known strategy as a model.	Social marketing	Show how E-CRM can be used to improve marketing positioning as explained in the article	Social marketing
	Write a C++ statement to declare a variable of type music Type name MyTune.	Computer programming	Sketch a prediction of the field lines for the arrangement of electrodes shown in Fig 2	Physics
**ANALYSIS**	What is the relationship between probability and statistical analysis?	Math	By comparing the map of the tectonic plates to the earthquake map, what inferences can you make?	Geology
	Analyze a work of art in terms of form, color and texture.	Art	Break down the components of a standard film camera and explain how they interact to make the machine work.	Physics
**SYNTHESIS**	Write a JAVA program to show the Overloading concept	Computer programming	Explain how the biological concept of symbiotic relationships could be used to help solve socially created problems like water pollution, overflowing garbage landfills, or homelessness.	Ecology
	Use your imagination to create a picture about the story. Then, add one new thing that was not in the story.	Literature	Develop a SQA Plan for a software development project which is defined in the attached document.	Software engineering
**EVALUATION**	Your advice has been sought to settle the following dispute in Company X. Referring to appropriate legal principles, write a short report advising the company on the best course of action to adopt.	Law	Conclude and support which economic system leads to a higher standard of living.	Economy
	"Don’t use public instance variables" is defensive programming techniques. discuss why it is good advice?	Computer programming	Examine the stated positions of both major political candidates with regard to a particular issue and state good reasons (based on principles discussed in class) for why one candidates position is more likely to be effective than the other’s.	Political science

### 3.2 Pre-processing

Performing pre-processing for unstructured data is highly recommended in any framework which uses machine learning [43] to reduce the unnecessary, duplicated, irrelevant, and noisy data. Therefore, pre-processing is implemented before the feature extraction phase and the classification process. However, in the pre-processing stage, the questions had to pass through several steps: normalization, tokenization, part-of-speech tagging, and stemming in order to refine and prepare them for the next stage.

The first step was the normalization process in which the unwanted data such as punctuation marks, numbers, and non-English characters will be eliminated. Then, all the words in the question will be converted into lowercase. In addition, stop-words are discarded from the question. In this step, not all stop-words will be eliminated from the question. The reason behind keeping some of the stop-words is that some questions contain important words, which can be considered as stop words in the default stop-words list, while these words have a significant impact in determining the level of the question. For example, the question ‘*Define transferable skill*’ belongs to *Knowledge level*, while the question ‘*In your own words*, *how would you define transferable skills*’ belongs to the *Comprehension level*. In case of eliminating the stop-words from the latter example, the question will be ‘*words*, *define transferable skills*’ where the following words will be deleted ‘*your*, *own*, *how*, *you*’, which means the question will look like *Knowledge level* question; these words can be effectively used in determining the class of question during classification process.

After performing normalization for the question, the tokenization will take place by splitting the question into individual words based on the whitespaces, known as tokens. Then the Stanford Part-of-Speech (POS) tagger (version 3.9.1) [44] tags the question words. The word will be labelled with a syntactic class such as noun (NN), verb (VB), adjective (JJ), and adverb (RB). Finally, the last step in pre-processing is stemming, the process of deleting the prefix and suffix in order to return the word into its original root. For example, *waits*, *waiting*, and *waited* after stemming will be *wait*. However, in order to perform stemming one of the most popular stemmers is used, which is the Porter stemmer in NLTK toolkit [45]. Unfortunately, the stemmer sometimes suffers from a problem of failure to return the correct root of some words e.g., *recalls*, *recalling* and *recalled* after stemming will be *recal* instead of *recall*. Although, in this case, it does not matter since similar terms are stored at the same index, which means during search and retrieval, the desirable word will be retrieved correctly. Moreover, POS tagging and the stemming will be used only with the TFPOS-IDF feature, and not word2vec as it will be discussed later.

### 3.3 Feature extraction

After pre-processing have been being applied, now the questions are ready for features extraction phase. Feature extraction is a process of transforming the raw input data into a meaningful set of features [46], in such a way that it can be understandable to the machine learning classifiers. Two features will be extracted. The aim of the first feature is to develop a weighting method to set the priority of words based on modified TF-IDF with Part-of-Speech (POS). Whereas the second feature is used to enhance the classification process, by extracting the semantic feature based on pre-trained word2vec. These two features will be combined to produce the vectors that will be used in the classification process.

#### 3.3.1 Modified TF-IDF (term weighting method TFPOS-IDF)

The Term Frequency (TF) is known as a local term weight, whereas the Inverse Document Frequency (IDF) known as a global term weight, is calculated using the following formulas:

$$TF(t,d)\;=\;\frac{c(t,d)}{\sum_{i}c(t_{i},d)}$$ (2)

$$IDF(t)\;=\;1+log(\;\frac{D}{d_{t}}\;)$$ (3)

TF-IDF(t,d)=TF(t,d).IDF(t) (4)

where *c(t*,*d)* indicates the occurrence of term *t* appears in document *d*, and the denominator $\sum_{i}c(t_{i},d)$ indicates the total number of terms in document *d*, *D* is the total number of documents in the dataset, and *d*_*t*_ is the number of the documents a term *t* appeared in. Many researchers [26–28, 36] tries to improve the performance of TF-IDF by proposing some modification to the original equation of TF-IDF. In the case of Bloom’s taxonomy verb plays an important role to determine the level of the question. Relying on the pre-defined list does not always guarantee a good performance, especially for words that appear in more than one level. Therefore, the proposed feature Term Frequency (TF)-Inverse Document Frequency (IDF) based on Part-Of-Speech (POS) is introduced known as TFPOS-IDF. The idea beyond this approach is inspired from [26]. In order to determine the suitable rank for word weight according to Bloom’s taxonomy, several cases have been examined. The experiments have been run for fifteen times using both dataset and three classifiers. Table 3 demonstrates the average of weighted F1-measure using KNN, LR, and SVM with the collected dataset. Table 4 displays the average of weighted F1-measure using KNN, LR, and SVM with the Yahya et al.'s (2012) dataset.

**Table 3:** Weighted F1-measure of different weight cases with collected dataset using KNN, LR and SVM.

Cases	w_1_	w_2_	w_3_	KNN	LR	SVM
CASE 1	VB	OTHERWISE	-	0.634	0.733	0.738
CASE 2	VB	NN	OTHERWISE	0.656	0.751	0.759
**CASE 3**	**VB**	**NN–JJ**	**OTHERWISE**	**0.659**	**0.756**	**0.764**
CASE 4	VB	NN-RB	OTHERWISE	0.647	0.739	0.749
CASE 5	VB	NN-JJ-RB	OTHERWISE	0.633	0.743	0.751
CASE 6	VB-WH	OTHERWISE	-	0.568	0.703	0.718
CASE 7	VB- WH	NN	OTHERWISE	0.621	0.698	0.652
CASE 8	VB- WH	NN-JJ	OTHERWISE	0.614	0.701	0.721
CASE 9	VB- WH	NN-RB	OTHERWISE	0.625	0.698	0.718
CASE 10	VB- WH	NN-JJ-RB	OTHERWISE	0.605	0.701	0.721
CASE 11	VB-WH-MD	OTHERWISE	-	0.574	0.687	0.715
CASE 12	VB-WH-MD	NN	OTHERWISE	0.610	0.687	0.713
CASE 13	VB-WH-MD	NN-JJ	OTHERWISE	0.606	0.694	0.717
CASE 14	VB-WH-MD	NN-RB	OTHERWISE	0.610	0.687	0.713
CASE 15	VB-WH-MD	NN-JJ-RB	OTHERWISE	0.619	0.694	0.717

**Table 4:** Weighted F1-measure of different weight cases with Yahya et al. (2012) dataset using KNN, LR and SVM.

Cases	w_1_	w_2_	w_3_	KNN	LR	SVM
CASE 1	VB	OTHERWISE	-	0.800	0.828	0.847
CASE 2	VB	NN	OTHERWISE	0.824	0.848	0.861
**CASE 3**	**VB**	**NN–JJ**	**OTHERWISE**	**0.834**	**0.856**	**0.866**
CASE 4	VB	NN-RB	OTHERWISE	0.817	0.834	0.841
CASE 5	VB	NN-JJ-RB	OTHERWISE	0.826	0.837	0.855
CASE 6	VB-WH	OTHERWISE	-	0.749	0.804	0.790
CASE 7	VB- WH	NN	OTHERWISE	0.766	0.812	0.796
CASE 8	VB- WH	NN-JJ	OTHERWISE	0.780	0.815	0.800
CASE 9	VB- WH	NN-RB	OTHERWISE	0.770	0.814	0.797
CASE 10	VB- WH	NN-JJ-RB	OTHERWISE	0.777	0.817	0.804
CASE 11	VB-WH-MD	OTHERWISE	-	0.747	0.805	0.799
CASE 12	VB-WH-MD	NN	OTHERWISE	0.762	0.815	0.803
CASE 13	VB-WH-MD	NN-JJ	OTHERWISE	0.770	0.816	0.808
CASE 14	VB-WH-MD	NN-RB	OTHERWISE	0.763	0.817	0.806
CASE 15	VB-WH-MD	NN-JJ-RB	OTHERWISE	0.764	0.816	0.808

where MD, WH, VB, NN, JJ, and RB stand for the model, wh-question word, verb, noun, adjective, and adverb, respectively. From the results shown in Table 3, the best result achieved with the collected dataset when verb assigned to w_1_, noun and adjective assigned to w_2_, and other POS assigned to w_3_. Similar experiments had been implemented using Yahya et al.'s (2012) dataset. Table 4 shows the average of weighted F1-measure using KNN, LR, and SVM with Yahya et al.'s (2012) dataset.

The findings stated from Tables 3 and 4, confirm that verbs are the most important words to determine the level of the question according to Bloom’s taxonomy. In addition, assigning a higher weight for wh-question words, or model along with verbs does not help to improve the performance of classification since these words appear in all levels. Furthermore, nouns often carry significant information in question and document classification [10]. That is obviously clear by comparing the results of Case 1 versus Case 2, Case 6 versus Case 7, and Case 11 versus Case 12. Giving noun some priority over other POS improves the performance of the classification. Lastly, it can be observed that the best result is obtained in Case 3. Which means adjective also carries a significant information in question classification based on Bloom’s taxonomy, since it improves the classification outcome.

Thus, in the case of question classification based on Bloom’s taxonomy cognitive domain, the highest priority will be given to verb by assigning w_1_. Moreover, from the experiments, the best result with both datasets and both classifiers are obtained when the noun and adjective are assigned to w_2_. Therefore, noun and adjective will be in the second rank, in which their weight will be less than the verbs but more significant than other POS. Lastly, other POS such as preposition, and pronoun will be assigned to the lowest weights. The method for assigning a weight for the term *t* is illustrated by Eq 5

$$w_{pos}\left(t\right)=\{\begin{array}{cc} w_{1} & if\;t\;is\;verb\\ w_{2} & if\;t\;is\;noun\;or\;adjective\\ w_{3} & otherwise \end{array}$$ (5)

where w_1_>w_2_>w_3_> 0, assume that the weights values for w_1_,w_2_, and w_3_ are w_1_ = 5, w_2_ = 3 and w_3_ = 1, as in [26] study. Now the new equation that represents TFPOS, i.e. the term frequency based on POS is:

$$TFPOS(t,d)\;=\;\frac{c(t,d)*w_{pos}(t)}{\sum_{i}\;c(t_{i},d)*w_{pos}(t_{i})}$$ (6)

finally, the TFPOS-IDF(t,d) is calculated for term *t* in document *d* by the following equation:

TFPOS-IDF(t,d)=TFPOS(t,d).IDF(t) (7)

The output of this phase is the sparse matrix, i.e. high-dimensional vectors. After calculating TFPOS-IDF the feature vectors will be normalized to prevent the numerical complexities during the calculation. L2 norm is used for normalization, which is the most popular norm. The representation of the L2 norm is demonstrated by the following equation:

$${\left\|\vec{v}\right\|}_{2}\;=\;(\sum_{i\;=\;1}^{n}{\left|v_{i}\right|}^{2}{)}^{\frac{1}{2}}$$ (8)

Now the feature vector representation is already normalized in which the sum of square values of a row is equal to one. For example, the values of each term in question “*Recall the main components of the flowchart*” after pre-processing and applying TF-IDF and TFPOS-IDF is shown in Table 5. The higher value indicates that it is a more relevant and important term in this document. For example, the TF-IDF’s value for recall is 0.774, but for the modified feature TFPOS-IDF is 0.878 since this word is a verb, it has a higher value, which denotes that recall appears more in this document. Whereas other words main, components and flowchart have a higher value in TF-IDF comparing to the modified feature TFPOS-IDF which reduces the values of these words, since it is not as important as verb.

**Table 5:** Example of weighting method using classical TF-IDF and modified TFPOS-IDF.

Tagged terms	recall / VB	main /JJ	components /NNS	flowchart/NN
Stemmed terms	recal	main	compon	flowchart
**TF-IDF**	0.774	0.581	0.194	0.161
**TFPOS-IDF**	0.878	0.440	0.146	0.122

#### 3.3.2 Word2vec

The first proposed feature TFPOS-IDF focuses on giving verbs higher priority over other words. Focuses on the verbs might not be enough. Therefore, it is worthwhile to try extracting another feature, which is the semantic feature word embedding; to the best of our knowledge has not been applied yet in the question classification based on Bloom’s taxonomy. Since the context of the question might carry an important information, this may help in determining the level of question.

This study used pre-trained word2vec, which trains approximately one hundred billion tokens using Google News dataset. The word vector for each word consists of floating-point values, and the meanings of these feature values are hard to explain, except it denotes that the words with similar meaning have a similar vector representation. For example, the top five words that have a similar vector to the word ‘*Examine*’ are ‘*Scrutinize*, *Investigate*, *Determine*, *Assess*, *Evaluate*’.

**Fig 3:**
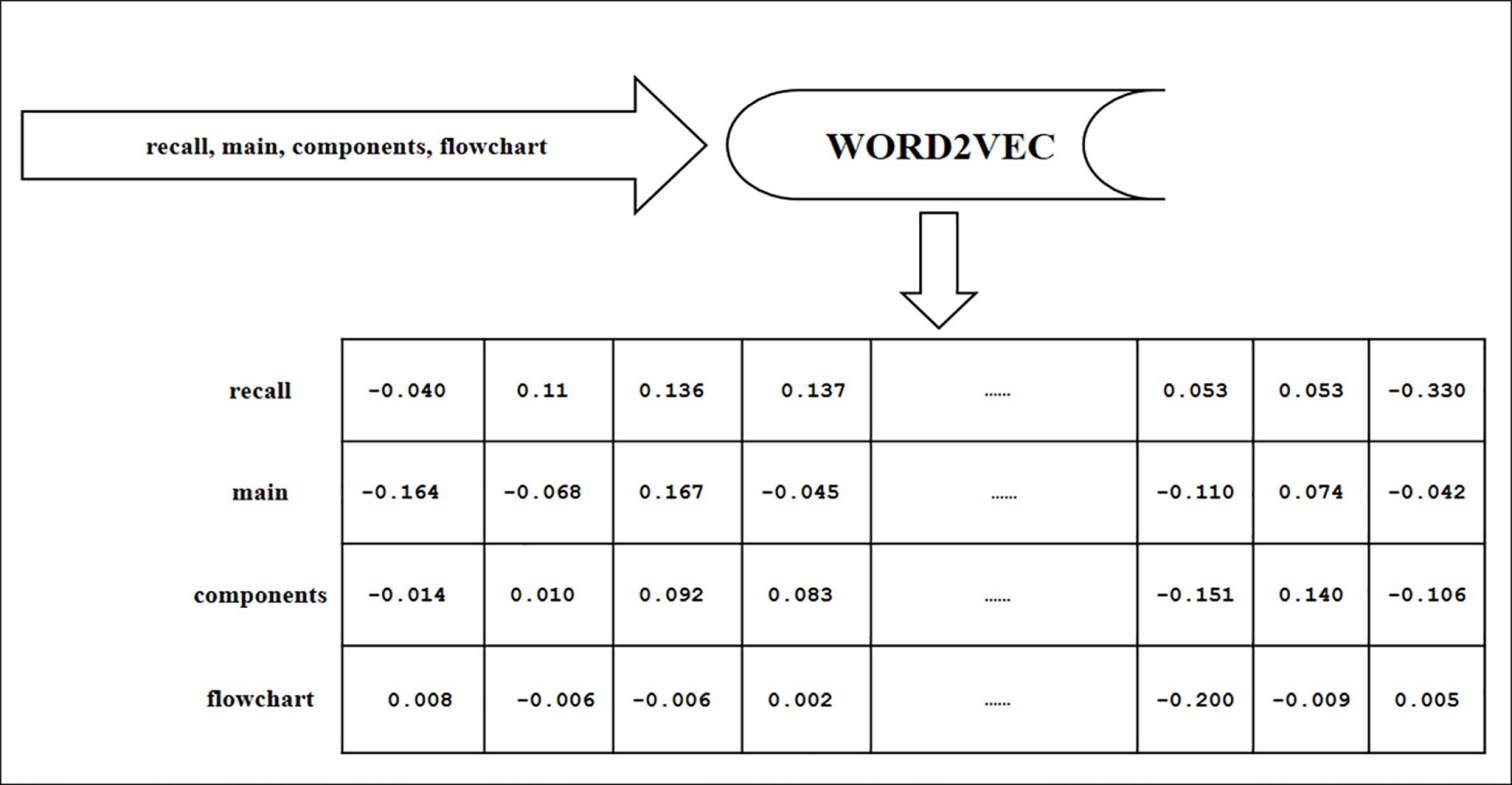
Example of converting question into a word vector.

Fig 3 shows an example of converting question into vector representation. The tokenized question that was produced after the pre-processing step, will be converted into the matrix of floating-point values with the size of 300 dimensions. Gensim library is used to retrieve the word vectors from the word2vec. Lastly, if the word does not exist on the word2vec, then vectors will be initialized by zeros.

As mentioned previously, the stemming process is performed only in the TFPOS-IDF step. The question word in this step is not stemmed. The reason behind keeping the word as it is without stemming, is that the pre-trained word2vec, i.e. (Google News) is not pre-processed before the process of learning and establishing the word vectors, and it builds the relationships between these vectors. For example, there is no word vector for the stemmed word ‘*recal*’. But on the other hand, there are word vectors for *‘recall’*, *‘recalling’*, and *‘recalls’* which are not totally equal but approximately have similar vectors. The strength of these features lies in producing similar word vectors for the words that have similar meanings, in low dimensional vectors representation. After retrieving the words vector for each question in the corpus, it is a time to combine them with the improved feature TFPOS-IDF.

#### 3.3.3 Combination of word2vec and TFPOS-IDF (W2VTFPOS-IDF)

**Fig 4:**
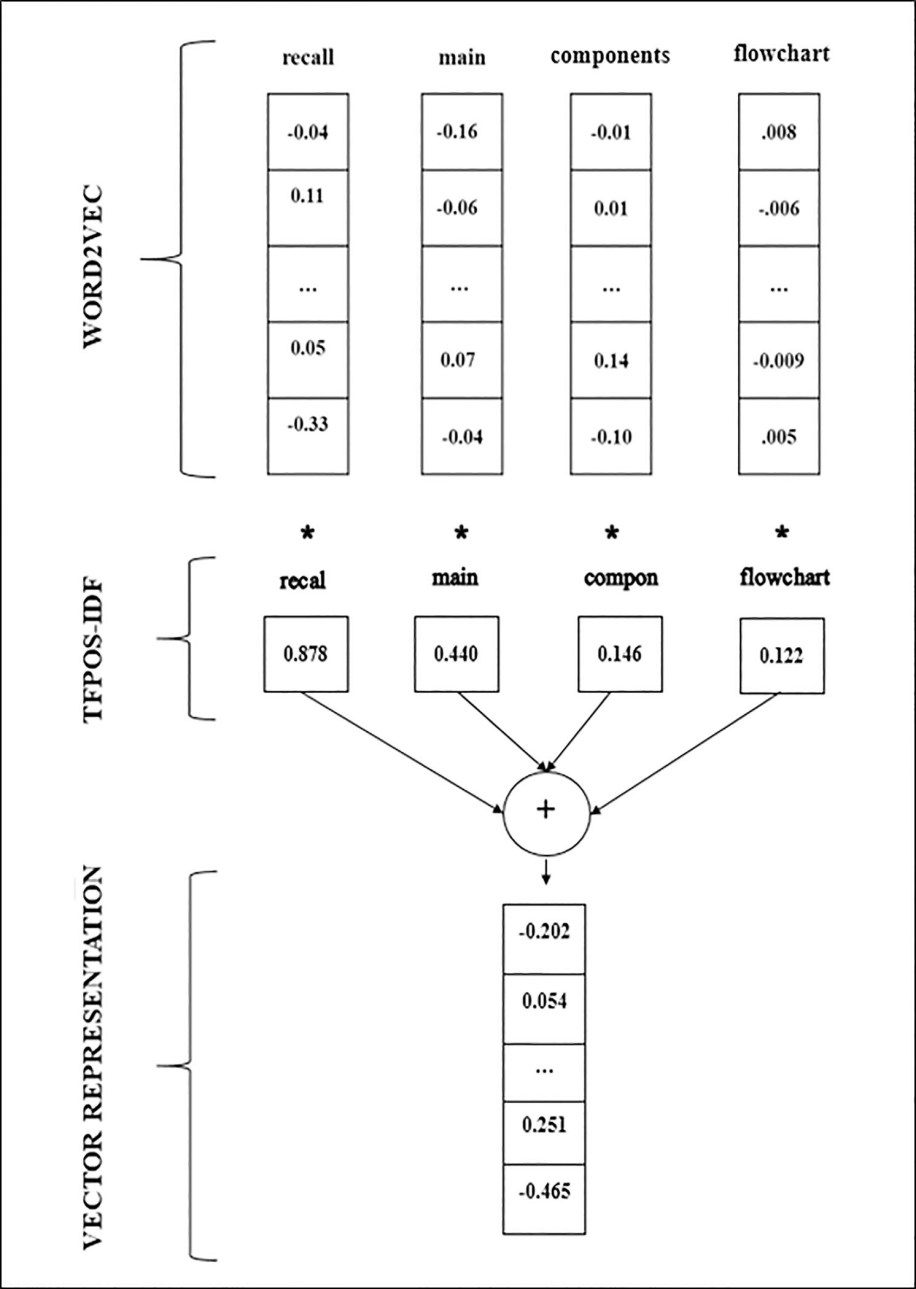
Example of combining word2vec with TFPOS-IDF.

The aim of this phase is to produce a question with high-quality feature vectors representation by combining the two proposed features, discussed before in the previous two sections. [32] used word2vec with TF-IDF to represent feature vector for question classification in the question answering system, by multiplying the term value of TF-IDF with the word vector of that term. Then after that, all the terms vector of the question is summed together to produce a single vector. This study uses the same approach as demonstrated in Eq (9).

$$Question\;Vector\;=\;\sum_{t\in d}^{}word2vec(t)*[TFPOS-IDF(t,d)]$$ (9)

where the term *t* belongs to the document *d*. The output of this phase is a dense vector, i.e. low-dimensional vector. The size of the vector is 300 dimensions since it is based on the pre-trained word2vec with the dimensions of 300. The advantage of representing question vector in the dense representation (low dimensional) over sparse representation (high dimensional) lies in decreasing the complexity of the classification process [36]. Fig 4 demonstrate an example of producing question vectors by combining word2vec with TFPOS-IDF.

Now the questions vectors W2V-TFPOSIDF are ready to be fed into the classification algorithms, which will be implemented in the next section.

### 3.4 Classification

Three of the most common supervised machine learning classification algorithms are used, which are the and K-Nearest Neighbour (KNN), Logistic Regression (LR), and Support Vector Machine (SVM).

#### 3.4.1 K-Nearest Neighbours (KNN)

K-Nearest Neighbours (KNN) is one of the simplest algorithms in machine learning. KNN performs well with low-dimensional vectors as shown in[36,47]. This algorithm is dominated by three crucial components [48]; (K) which is the number of nearest neighbours, the labelled training set, and how to estimate the distance among the points.

The way K-NN behave is by looking to the K nearest neighbours among the labelled training questions in which the similarity between a test question and K training questions is calculated to predict the class of the test question. The class or the category of the test question is assigned based on the highest score (majority class) of the K training questions. For example, for the Question X, if the algorithm found five nearest neighbours to X in which one belongs to the Knowledge class, one belongs to the Analysis class, two belongs to the Application class, and one belongs to the Evaluation class, then the question X is assigned to the Application class.

#### 3.4.2 Logistic Regression (LR)

Logistic Regression classifier is based on logistic function, also known as the sigmoid function, which has been used in several text classification applications [49,50] and shows significant results. The idea behind it is to find the relationship among features and specific output. Logistics regression algorithm applied one versus all approach for the multiclass classification task. For example, to predict the question class, six binary classification problem are considered i.e. whether the class is knowledge, comprehension and so on with all classes. Following this, the Maximum Likelihood Estimation is used to assign the predicted class and it is implemented by the following equation [51]:

$$P{\left(c|x\right)}^{}\;=\;\frac{e^{(\sum_{i\;=\;1}^{N}f_{i}(c,x)w_{i})\;}}{\sum_{c\prime \epsilon C}e^{(\sum_{i\;=\;1}^{N}f_{i}(c\prime ,x)w_{i})\;}}$$ (10)

#### 3.4.3 Support Vector Machine (SVM)

Support Vector Machine (SVM) was proposed by [52] at AT&T Bell Laboratories. It is widely used in text classification problems [53]. The idea behind SVM is to find an appropriate hyperplane that best split-up the two sets of data from each other, by maximizing the margin between the hyperplane and the set of data points nearest to it. In-text classification SVM with linear kernel perform well [54]. Therefore, this study will use it.

Given *N* data points from questions in the training set ${{\left\{x_{i},y_{i}\right\}}_{i\;=\;1}^{N}}_{}$, where *x*_*i*_ is the i-th input features (i.e. pattern), and *y*_*i*_ is the i-th output class label. The SVM linear kernel equation can be written as the following [55]:

$$y(x)\;=\;sign[\sum_{i\;=\;1}^{n}\propto_{i}y_{i}\;\theta (x,x_{i})+b]$$ (11)

where n is the number of support vectors, x_i_ is the pattern of i-th question, y_i_ is the class label of the i-th question, and *θ*(*x*,*x*_*i*_) is linear kernel function, which can be written as:

$$\theta (x,x_{i})\;=\;x_{i}^{T}x$$ (12)

### 3.5 Evaluation metrics

To measure the effectiveness of the proposed model, the weighted recall, precision and F1-mueasre will be calculated. In order to define these metrics, the terms True Positive (TP), False Positive (FP) and False Negative (FN) that must be introduced. TP is the occurrence of instances a classifier correctly classified to the suitable class. FP is the occurrence of instances incorrectly classified. FN is the occurrence of instances that have not been classified. The value of recall, precision, and F1-measure varies from 0 to 1. The closer value to zero of recall, precision, and F1-measure implies the poor performance. Whereas, the closer value to 1 denotes a good performance.

Recall metric measures the perfection of classifiers by computing this formula

$$Recall\;=\;\frac{TP}{TP+FN}$$ (13)

Precision metric measures the fineness of classifiers by calculating this equation

$$Precision\;=\;\frac{TP}{TP+FP}$$ (14)

Finally, the F-measures or also known as F-score, is calculated by

$$F_{\beta }-measure\;=\;\frac{\left(1+\beta^{2})*(Recall\;\times Precision\right)}{\left(\beta^{2}\;*\;Recall+Precision\right)}$$ (15)

When the value of *β* = 1 that represents F1-measure, where the equation will be:

$$F_{1}-measure\;=\;\frac{2*(Recall\;\times Precision)}{\;Recall+Precision}$$ (16)

Since the training data and test data are randomly selected, and usually imbalanced, the weighted recall, precision, and F1-measure will be calculated. Scikit-learn defined the term weighted “*Calculate metrics for each label*, *and find their average*, *weighted by support (the number of true instances for each label)*. *This alters ‘macro’ to account for label imbalance; it can result in an F-score that is not between precision and recall*.*”*.

The equations for calculating the weighted precision, weighted recall and weighted F1-measure are:

$$Weighted\;Recall\;=\;\frac{\sum_{i\;=\;1}^{n}\left({recall}_{i}*\;{support}_{i}\right)}{\sum_{i\;=\;1}^{n}{support}_{i}}$$ (17)

$$Weighted\;Precision\;=\;\frac{\sum_{i\;=\;1}^{n}\left({Precision}_{i}*\;{support}_{i}\right)}{\sum_{i\;=\;1}^{n}{support}_{i}}$$ (18)

$$Weighted\;F1-measure\;=\;\frac{\sum_{i\;=\;1}^{n}\left({F1-measure}_{i}*\;{support}_{i}\right)}{\sum_{i\;=\;1}^{n}{support}_{i}}$$ (19)

## 4. Experiment setting

In order to evaluate the effectiveness of the proposed model, several experiments had been conducted, with three features and three classifiers. The first feature was the traditional statistical feature TF-IDF. The second feature was the modified feature TFPOS-IDF. The purpose of mentioning the traditional feature TF-IDF was to compare it with the improved feature TFPOS-IDF, in order to check whether the improvement is significant or not. The third feature W2V-TFPOSIDF is word2vec, which was weighted by the improved feature TFPOS-IDF. All these features were fed into three classifiers KNN, LR, and SVM with a linear kernel, which were implemented using Scikit-learn library in Python with the default settings. The default value of K in the KNN classifier is five, and the default value of the penalty is l2 in LR. The default setting of the parameter C in SVM is 1. However, the experiments were run 15 times; during each run, the training and testing set were randomly picked up. The dataset was divided into 80% training set and 20% test set, for the first dataset, the size of the training set was 112 and 29 for the testing set. The size of the training set in the second dataset is 480, and the testing set is 120.

### 4.1 Result of KNN

This section discusses the experiments result that has been obtained from the collected dataset and Yahya et al. (2012) dataset using the KNN classifier in terms of weighted recall, precision, and F1-measure for each Bloom class. The experiments measured the performance of KNN with three features TF-IDF, TFPOS-IDF, and W2V-TFPOSIDF.

It was observed from the result demonstrated in Table 6 that the modified feature TFPOS-IDF performs better than the traditional feature TF-IDF from all aspects. In addition, the proposed features W2V-TFPOSIDF outperform the improved feature by 5.2% in terms of F1-measure. On the other hand, the score of recall, precision, and F1-measure for the application class with all features are low compared to other classes. This is due to the small number of instances which belongs to the application level in the collected dataset. Therefore, the learning algorithm KNN misclassified the questions that belong to the application level.

**Table 6:** Results of using KNN with TF-IDF, TFPOS-IDF, W2V-TFPOSIDF for the collected dataset.

Cognitive Level	TF-IDF			TFPOS-IDF			W2V-TFPOSIDF		
	Recall	Precision	F1-measure	Recall	Precision	F1-measure	Recall	Precision	F1-measure
Knowledge	0.781	0.800	0.777	0.794	0.887	0.822	0.782	0.947	0.832
Comprehension	0.889	0.778	0.819	0.920	0.783	0.837	0.857	0.617	0.691
Application	0.134	0.316	0.173	0.134	0.341	0.183	0.307	0.580	0.375
Analysis	0.742	0.464	0.553	0.741	0.526	0.601	0.707	0.798	0.729
Synthesis	0.691	0.660	0.657	0.801	0.694	0.729	0.881	0.777	0.808
Evaluation	0.464	0.742	0.554	0.559	0.749	0.627	0.655	0.825	0.694
**Weighted Average**	**0.632**	**0.651**	**0.611**	**0.683**	**0.687**	**0.659**	**0.724**	**0.773**	**0.711**

Based on the results demonstrated in Table 7, it can be observed that the classifier learnt more about Bloom’s cognitive levels using the modified feature TFPOS-IDF, since the performance has increased from all aspects. The classification results of using the proposed feature W2V-TFPOSIDF outperforms the modified feature TFPOS-IDF. The result with this dataset is better than the obtained result from the collected dataset, since the size of Yahya et al. (2012) dataset is larger than the collected dataset. Moreover, the number of the questions in Yahya et al. (2012) dataset is distributed evenly over all classes, unlike the collected dataset.

**Table 7:** Results of using KNN with TF-IDF, TFPOS-IDF, W2V-TFPOSIDF for the Yahya et al. (2012) dataset.

Cognitive Level	TF-IDF			TFPOS-IDF			W2V-TFPOSIDF		
	Recall	Precision	F1-measure	Recall	Precision	F1-measure	Recall	Precision	F1-measure
Knowledge	0.946	0.735	0.824	0.987	0.798	0.880	0.933	0.866	0.895
Comprehension	0.796	0.814	0.800	0.812	0.865	0.834	0.880	0.919	0.896
Application	0.660	0.826	0.734	0.820	0.870	0.846	0.869	0.847	0.856
Analysis	0.875	0.687	0.767	0.903	0.837	0.865	0.920	0.815	0.864
Synthesis	0.686	0.795	0.730	0.725	0.788	0.750	0.788	0.812	0.794
Evaluation	0.634	0.862	0.723	0.755	0.917	0.820	0.729	0.947	0.817
**Weighted Average**	**0.766**	**0.786**	**0.763**	**0.835**	**0.846**	**0.834**	**0.855**	**0.866**	**0.854**

### 4.2 Result of LR

This section discusses the results of the experiment that have been obtained with the LR classifier in terms of the weighted recall, precision, and F1-measure for each Bloom class. Table 8 demonstrates the results of the collected dataset. Table 9 shows the results of Yahya et al. (2012) dataset.

**Table 8:** Results of LR with TF-IDF, TFPOS-IDF, W2V-TFPOSIDF for the collected dataset.

Cognitive Level	TF-IDF			TFPOS-IDF			W2V-TFPOSIDF		
	Recall	Precision	F1-measure	Recall	Precision	F1-measure	Recall	Precision	F1-measure
Knowledge	0.833	0.898	0.851	0.833	0.924	0.864	0.885	0.960	0.910
Comprehension	0.873	0.798	0.814	0.937	0.897	0.907	0.857	0.886	0.860
Application	0.077	0.308	0.121	0.154	0.596	0.241	0.327	0.726	0.419
Analysis	0.759	0.592	0.645	0.810	0.646	0.695	0.948	0.844	0.882
Synthesis	0.910	0.701	0.782	0.970	0.747	0.835	0.930	0.825	0.861
Evaluation	0.655	0.823	0.709	0.750	0.917	0.811	0.893	0.883	0.879
**Weighted Average**	**0.722**	**0.712**	**0.688**	**0.779**	**0.802**	**0.756**	**0.834**	**0.860**	**0.823**

**Table 9:** Results of LR TF-IDF, TFPOS-IDF, W2V-TFPOSIDF for Yahya et al. (2012) dataset.

Cognitive Level	TF-IDF			TFPOS-IDF			W2V-TFPOSIDF		
	Recall	Precision	F1-measure	Recall	Precision	F1-measure	Recall	Precision	F1-measure
Knowledge	0.907	0.807	0.850	0.997	0.826	0.901	0.987	0.917	0.950
Comprehension	0.803	0.913	0.852	0.806	0.930	0.859	0.858	0.970	0.908
Application	0.728	0.905	0.804	0.797	0.898	0.843	0.858	0.876	0.865
Analysis	0.907	0.733	0.807	0.927	0.864	0.893	0.944	0.939	0.940
Synthesis	0.742	0.863	0.793	0.777	0.856	0.810	0.845	0.835	0.834
Evaluation	0.808	0.765	0.779	0.840	0.831	0.829	0.868	0.868	0.865
**Weighted Average**	**0.814**	**0.833**	**0.814**	**0.857**	**0.868**	**0.856**	**0.893**	**0.901**	**0.894**

Tables 8 and 9 show the performance of the LR classifier with both datasets in which the W2V-TFPOSIDF feature produces a better result than TFPOS-IDF and TF-IDF.

### 4.3 Result of SVM

This section discusses the results of the experiment that have been obtained with the SVM classifier in the terms of the weighted recall, precision, and F1-measure for each Bloom class using both dataset; collected dataset, and Yahya et al. (2012) dataset.

As shown in Table 10 the SVM classifier with the modified feature TFPOS-IDF outperforms the traditional feature TF-IDF in terms of all the evaluation metrics recall, precision and F1-measure, as well as W2V-TFPOSIDF which outperform the two other features.

**Table 10:** Results of SVM with TF-IDF, TFPOS-IDF, W2V-TFPOSIDF for the collected dataset.

Cognitive Level	TF-IDF			TFPOS-IDF			W2V-TFPOSIDF		
	Recall	Precision	F1-measure	Recall	Precision	F1-measure	Recall	Precision	F1-measure
Knowledge	0.834	0.990	0.895	0.847	1.000	0.908	0.885	0.978	0.920
Comprehension	0.952	0.827	0.876	0.936	0.873	0.897	0.904	0.890	0.892
Application	0.153	0.538	0.229	0.173	0.596	0.261	0.462	0.629	0.507
Analysis	0.862	0.535	0.645	0.896	0.540	0.663	0.966	0.865	0.905
Synthesis	0.920	0.780	0.837	0.970	0.810	0.877	0.881	0.850	0.855
Evaluation	0.608	0.817	0.679	0.701	0.939	0.779	0.870	0.859	0.853
**Weighted Average**	**0.750**	**0.770**	**0.724**	**0.786**	**0.817**	**0.764**	**0.844**	**0.856**	**0.837**

The detailed results of using SVM classifier with Yahya et al. (2012) dataset have been shown in Table 11, where TFPOS-IDF outperforms TF-IDF, and W2V-TFPOSIDF Superior both features.

**Table 11:** Results of SVM TF-IDF, TFPOS-IDF, W2V-TFPOSIDF for Yahya et al. (2012) dataset.

Cognitive Level	TF-IDF			TFPOS-IDF			W2V-TFPOSIDF		
	Recall	Precision	F1-measure	Recall	Precision	F1-measure	Recall	Precision	F1-measure
Knowledge	0.980	0.841	0.902	0.987	0.893	0.935	0.993	0.929	0.961
Comprehension	0.799	0.925	0.854	0.816	0.932	0.868	0.871	0.945	0.905
Application	0.766	0.853	0.803	0.839	0.853	0.845	0.880	0.853	0.864
Analysis	0.914	0.788	0.845	0.938	0.910	0.921	0.961	0.929	0.943
Synthesis	0.714	0.868	0.778	0.791	0.831	0.805	0.841	0.850	0.841
Evaluation	0.797	0.756	0.771	0.825	0.823	0.819	0.829	0.915	0.866
**Weighted Average**	**0.828**	**0.839**	**0.826**	**0.867**	**0.874**	**0.866**	**0.897**	**0.902**	**0.897**

Based on the results from Tables (6–11), it can be concluded that LR and SVM approximately produce similar results, and achieve better performance compared to KNN in classifying questions from different domains in accordance with Bloom’s taxonomy, using both datasets.

## 5. Statistical test

The statistical test, the t-test can be used to decide whether two samples of data are significantly different from each other. In order to check the significance of the first modified feature TFPOS-IDF and the second proposed feature W2V-TFPOSIDF, the weighted F1-measure values of each run were used. This section discusses the results of the significant test in two phases, using both the datasets and the three classifiers KNN, LR and SVM. In the first phase, the first modified feature TFPOS-IDF was checked against the traditional feature TF-IDF, to check if the proposed improvement was significant or otherwise. The second phase, the TFPOS-IDF was checked against the W2V-TFPOSIDF to check the significance of adding TFPOS-IDF to the word2vec.

A two-sample t-test of the null hypothesis was used. The null hypothesis meant that the value of the hypothesized mean difference was equal to zero, which assumed that there was no difference between the two samples. The value of alpha P-value was equal to 0.05. The idea behind t-test was to check if the obtained value of alpha was less than the assumed one (i.e. P-value = 0.05), if so then the null hypothesis would be rejected, which means there was a significant difference between the two samples. Otherwise, if the P-value was greater than or equal to the assumed one, then the null hypothesis was accepted, which meant that there was no significant difference between the two samples. The alpha values of the t-test are shown in Table 12.

**Table 12:** Alpha values of t-test.

Classifier	Dataset	TF-IDF vs. TFPOS-IDF	TFPOS-IDF vs. W2V-TFPOSIDF
KNN	Collected Dataset	0.002056	0.041744
	Yahya et al. (2012) Dataset	9.95E-06	0.049986
LR	Collected Dataset	0.001163	0.006075
	Yahya et al. (2012) Dataset	1.03E-05	6.63E-06
SVM	Collected Dataset	0.001089	0.00065
	Yahya et al. (2012) Dataset	6.34E-05	5.61E-05

As previously mentioned, if the P-value of the t-test is less than 0.05, then there is a significant difference between the two samples. Therefore, from the results shown in Table 11, it is clear that in all cases, the modified feature TFPOS-IDF is statistically significant compared to the classical one TF-IDF. In addition, the proposed feature W2V-TFPOSIDF is statistically significant compared to the modified feature TFPOS-IDF.

## 6. Conclusion

In conclusion, this paper proposed a method to classify open-domain questions based on Bloom’s taxonomy cognitive domain. In order to check the robustness of the proposed model, two datasets with three classifiers have been examined. In addition, the statistical test shows that there is a significant improvement between TF-IDF versus TFPOS-IDF and TFPOS-IDF versus W2V-TFPOSIDF. This method can be useful for educators and lecturers to analyze the exam question to fulfill the requirement for the different levels of education such as Bachelor’s degree or Master’s level. For example, the exam paper at a Master’s level usually contains the higher thinking order questions more than the lower thinking order. In addition, it can be effectively used throughout different kinds of applications such as automatic test generation systems, intelligent tutoring systems and even more in serious game such as in [56]. Nevertheless, further study could improve, enhance or extend this work by expanding the dataset through adding different kinds of questions. The dataset used in this study only consists of open-ended questions, in which there are neither multiple choices nor true or false questions. Most of the previous studies are conducted with an English dataset where there is a lack of studies in other languages. Since there is no public benchmark dataset that contains real exams questions labeled based on Bloom’s taxonomy, providing one would be beneficial to relevant researchers in this area.

## Supporting information

S1 File Collected dataset. (DOCX)

S2 File Yahya et al. (2012) dataset. (DOCX)
