# Supplementary material for: Question classification based on Bloom’s taxonomy cognitive domain using modified TF-IDF and word2vec
Source: PLoS One. 2020 Mar 19;15(3):e0230442. doi: 10.1371/journal.pone.0230442 (PMC7081997; doi:10.1371/journal.pone.0230442)
Supplement: S1 File — (DOCX) [file pone.0230442.s001.docx]

***Note:*** *This dataset collected from several websites, books and previous work^^[[1]](#footnote-1)^^, it consists of 141 questions, distributed as follows: Knowledge Level (26), Comprehension Level (23), Application Level (15), Analysis Level (23), Synthesis Level (30) and Evaluation Level (24).*

**Knowledge**

1. List two reference parameters in the setHour function
2. Explain briefly the meaning of the following terms: Demography, Consumer sales promotion , and Media mix
3. Label the parts of the diagram
4. Based on the above dataType class, list all the function members
5. Define morphology
6. Define Method in JAVA.
7. Define Inheritance concept
8. What is Encapsulation ?
9. Name the authors of...?
10. List the fractions you know and can show….
11. List the __________________ in descending order
12. List the attributes of the following shape.
13. List two local variables in the main function
14. List the data members
15. List important information you can remember from lesson….
16. Make a list of the main events of the story.
17. Memorize and recall the periodic table
18. Memorize the multiplication facts
19. Memorize the meaning of …
20. Name three 19th-century women English authors.
21. Name all the characters in the story.
22. Name five cities in US.
23. Recall the main components of the flowchart
24. Recall four facts from the story
25. What do you recall ...?
26. What is the definition of the following terms…

**Comprehension**

1. Can you explain what is happening . . . what is meant . . .?
2. Class Facility is an abstract class. Explain what this means
3. Draw a diagram explaining how air pressure affects the weather.
4. Describe how interest rates affect the economy
5. Explain what is happening in the first picture of the story.
6. Explain why the story has the title that it does.
7. Explain how the heart is like a pump.
8. Explain what is happening...
9. Explain what is meant.
10. Explain in one sentence what the method incFromN does
11. How would you explain...?
12. How would you express ________________?
13. How can you describe _____________?
14. How would you classify the type of ...?
15. How can you explain what is meant ...?
16. Draw a flowchart to accept a number and output its factorial.example 1: input 5 -> 5! = 5x4x3x2x1 -> output 120
17. Identify the key points in the text
18. Identify three mistakes from the passage and correct them.
19. State in your own words ...
20. State in one word ...
21. Put in your own words…
22. What is the difference between atomic and molecular mass?
23. In your own words, tell what the story is about.

**Application**

1. Design or sketch a marketing strategy for your product using a known strategy as a model.
2. Sketch an experiment to see how plants grow in different kinds of soil.
3. Sketch a diagram which shows these fractions or take photographs of the fractions.
4. Demonstrate and illustrate measures of central tendency and dispersion, write a journal entry.
5. Interpret the graph and state how many trees were cut down to produce paper.
6. How does the law of supply and demand explain the current increase in the price of fruit?
7. Predict what would happen if ...
8. Write a textbook about this topic for others.
9. Write what you might have done.
10. Write an explanation about this topic for others
11. Write a C++ statement to declare a variable of type musicType name MyTune.
12. Draw an illustration of the linked list after the execution of each of the statement below. (Use the original illustration for each question).
13. Write the definition of displaySize() method for each class
14. Suggest a way to deal with this scenario
15. Choose the best statements that apply ...

**Analysis**

1. Analyze the selected information.
2. Can you discriminate the difference parts . . . ?
3. Discriminate the pros and cons of ____.
4. Analyze the following questionnaire.
5. How can you categorize ____ according to...?
6. How can you distinguish the different parts of_____?
7. How can you differentiate between...?
8. Discriminate the different parts of the story (introduction, development, climax, resolution.)
9. Analyze a distinction. State the point of view of . . .
10. Categorize the story into parts and think of a good title for each of the parts.
11. Analyze a work of art in terms of form, color and texture.
12. Trace the contents of matrix from the following statements int matrix[3][2];int j, k;for (j = 0; j < 3; j++)for (k =h 0; k < 2; k++)matrik[j][k] = j + k;
13. Trace the value of alpha after the following code executes. int alpha[5];int j;alpha[0] = 5;for (j = 1; j < 5; j++){if(j % 2 == 0)alpha[j] = alpha[j – 1] + 2;else alpha[j] = alpha[j – 1] + 3;}
14. What distinctions can be made about...and...?
15. What is the relationship between probability and statistical analysis?
16. What is the analysis of ______________?
17. Draw a diagram to show the memory configuration after the following statements ....
18. What has the author used to create this effect
19. Compare and contrast the two items and produce a summary of their similarities and differences
20. Develop a set of criteria for distinguishing between a good and bad example of this.
21. What evidence can you find to suggest that people should be regarded as the most important resource of an organisation?
22. While the futures market is too risky for most investors, this risk creates the opportunities for large returns. Discuss.
23. What evidence can you list for...

**Synthesis**

1. Create several different strategies to solve a mathematical problem.
2. Can you propose an alternative plan to . . . ?
3. Can you develop method to handle the following case . . . ?
4. Can you propose a model that would change . . . ?
5. Can you think of an original way for collect and develop the . . . ?
6. Develop a menu for a new healthy foods restaurant
7. Design a scientific study to test the effect of different kinds of music on hens’ egg production.
8. Design a building to house your study.
9. Design a record, book or magazine cover for...
10. Design a new monetary system or an experiment for establishing.
11. Create several scientific hypotheses to …
12. Propose an alternative way to solve …
13. Propose a set of alternatives for reducing dependence on fossil fuels that address both economic and environmental concerns.
14. Rewrite the story from an animal’s point of view.
15. Rewrite the story briefly, but change someone or something in it. (For example, substitute a dog for a wolf in The Three Little Pigs).
16. Use your imagination to create a picture about the story. Then, add one new thing that was not in the story.
17. What hypotheses can you develop based on the data ? Why ?
18. Write a journal from the point of view of mountaineer.
19. Write about your feelings in relation to...
20. Write a TV show play, puppet show, role play, song or pantomime about..
21. Write a poem about the story.
22. Write 5 new titles for the story that would give a good idea of what it was about
23. Write another ending to the story that is different from the author’s ending.
24. Write a JAVA program to show the Overloading concept
25. Write a program that uses nested loops to print the following output:
26. Write a program to calculate the SQRT for Array elements, you may use the Math class function (sqrt), the Array should include 5 Integer elements. The output should be the array elements along with their SQRT value.
27. Create an advertising campaign
28. Construct an alternative way to ...
29. Devise a different way of using X
30. Merge the different ideas into a single solution.

**Evaluation**

1. Can you defend your position about ... ?
2. Can you assess the value or importance of . . . ?
3. What limitation does X have?
4. Develop a proof ... and justify each step ...
5. "Don’t use public instance variables" is defensive programming techniques. discuss why it is good advice?
6. How would you justify . . . ?
7. Judge the value of... What do you think about...?
8. Judge how well a project meets the criteria of a rubric.
9. Judge the validity of arguments for and against astrology
10. Justify the concept of inheritance and give the sample of code to illustrate your answer.
11. Justify the object oriented programming concept
12. Prepare a case to present, and evaluate your view about ...
13. Prepare a list of criteria to judge ... Evaluate expressions
14. Rate the following recommendations and set the priority _____.
15. What data was used to evaluate ____________?
16. What judgments can you make about...?
17. What criteria would you use to assess...?
18. What criteria would you use to evaluate if your answer is correct?
19. What would you cite to defend the actions . . . ?
20. Outline how class ArrayList could be implemented using an array
21. Can you justify the decisions you have made ?
22. How would you rank the items given the criteria ?
23. Your advice has been sought to settle the following dispute in Company X. Referring to appropriate legal principles, write a short report advising the company on the best course of action to adopt.
24. What criticisms could you make ?

1. <https://academicamentoring.com/wp-content/uploads/2017/01/How-to-use-Blooms-Taxonomy-in-the-classroom.pdf> , <https://www.monash.edu/rlo/study-skills/preparing-for-exams/understanding-exam-questions> , <https://s3.amazonaws.com/scschoolfiles/725/bloomstaxonomy1.pdf> , (Haris & Omar 2015) , and others. [↑](#footnote-ref-1)
