# Supplementary material for: Question classification based on Bloom’s taxonomy cognitive domain using modified TF-IDF and word2vec
Source: PLoS One. 2020 Mar 19;15(3):e0230442. doi: 10.1371/journal.pone.0230442 (PMC7081997; doi:10.1371/journal.pone.0230442)
Supplement: S2 File — (DOCX) [file pone.0230442.s002.docx]

**Knowledge**

1. About what proportion of the population of the US is living on farms?
2. Correctly label the brain lobes indicated on the diagram below
3. Define compound interest.
4. Define four types of traceability.
5. Define mercantilism.
6. Define stream bank, floodplain and substrate.
7. Define the meaning of the Olympic Motto.
8. Draw and label a diagram of a typical stream.
9. Draw the basic outline of a page of notes using the Cornell Method?
10. Draw the block diagram of the central processing unit
11. Draw the flow chart of the PNK system
12. How many instructions in the instruction set of RISC computer?
13. How many kilometers from your home to the university
14. How many loop statement available in c++?
15. How many states are there in USA
16. How many years does this animal live?
17. How much data can be stored in the cach memory
18. How much did he pay for his car
19. How much is your weight
20. How much time did he took to build a house
21. How much water does an elephant drink
22. Identify a phrase as a simile
23. Identify a property (commutative, additive, etc(
24. Identify fractions from a pictorial representation
25. Identify ordinal positions
26. Identify the correct definition of osmosis.
27. Identify the five major prophets of the Old Testament.
28. Identify the part of a eukaryotic cell.
29. Identify the standard peripheral components of a computer.
30. Identify which word begins with the same sound as the s in sun (Knowledge of letter s)
31. in which continent does the state of suriname fall
32. in which countries is arabic language spoken
33. In Which part of the world does he live
34. Label any Olympic sporting apparatus with design features.
35. Label the parts of the following insects: Grasshopper
36. Label the parts of the microscope shown on the right
37. List four types of clauses mentioned in the article
38. List reserved words in C programming.
39. List the characteristics peculiar to the Cubist movement.
40. List the levels in Bloom's Taxonomy.
41. List the procedure to draw a mixed potential or Evans' diagram
42. List the steps involved in titration.
43. List three characteristics that are unique to the Cubist movement.
44. List three predominant economic systems that exist
45. Look at the symbol. What is the symbol’s purpose on a map?
46. Make a list of facts you learned from the story.
47. Name 5 steps that follow the taking of notes (within 24 hours or less)?
48. Name the artist who painted the Mona Lisa.
49. Name the food groups and at least two items of food in each group.
50. Name the four major food groups.
51. Name the main characters in the story.
52. Name the property that states that
53. Please label the diagram below:
54. Recall basic math facts
55. Recall locations of a historical events
56. Recall the definition of the formula datatype from your assignment 2.
57. Recall the equation for the Ideal Gas Law.
58. Recall the following pictoral example of an implementation of some queue operations
59. recall the supply voltage constraints
60. Recite a policy of immigration in Europe .
61. Recite the poem "Auto Wreck".
62. Recite the principles of the Data Protection Act.
63. Recite the the Gaussian law
64. State four attributes of well engineered software.
65. State how to find a word in a dictionary
66. State the characteristics of each type of corrosion testing studied
67. State the definition of application in Bloom's Taxonomy.
68. State the formula for the area of a circle
69. State the guidelines to design an anodic protection system
70. State the pattern for expressing time in French.
71. State the rule for balls and strikes in baseball.
72. State the rule for using a semicolon in a sentence.
73. What are major facts about particular cultures and societies
74. What are the major products and exports of countries
75. What are the representational symbols on maps and charts
76. What are the structure or functions of Congress
77. What are the units of corrosion penetration rates? Relate these to Faraday's law
78. What does ICT stands for.
79. What is your family name
80. What is a global variable?
81. What is a textual index
82. What is the setting of To Kill a Mockingbird?
83. What is the title of the first movie you saw
84. What season are we in right now?
85. when did saudi arabia start using internet
86. when did the first saudi radio station open
87. When did the second world war happen
88. When did you born ?
89. when was the first airport in the yemen built
90. when was the UN formed
91. Which of the following is the method for determining the volume of a cylinder?
92. Which of the following item does not belong to the set ?
93. Which of the two liquids is hypotonic
94. which part of a data packet is used by a router to determine the destination network
95. Which technology is used by laser printer
96. Who is the first president of USA
97. who is the founder of the kingdom of saudi Arabia
98. who was the first man on the moon
99. who was the first to compose in the arts of makamat
100. Who was the writer of the Hamilton

**Comprehension**

1. Compare Calliope with Howie. Use the word bank.
2. Compare historical events to contemporary situations
3. Complete an analogy (analogy tasks are inference tasks)
4. Describe 4 types of coupling in software design.
5. Describe how Phillip and Timothy survived on the Cay.
6. Describe in prose what is shown in graph form.
7. Describe in your own words how to borrow a book from the library.
8. Describe in your own words how to copy text from one program into another.
9. Describe in your own words what happens when a stream's velocity slows.
10. Describe in your own words what is meant by a sprained ankle.
11. Describe nuclear transport to a lay person.
12. Describe the Pareto Principle in statistical software quality assurance.
13. Describe what goes in each of the four areas on the first page of notes
14. Describe what took place as the Hato was sinking.
15. Describe how the linear polarization method can yield corrosion rates
16. Describe the major clinical differences between visceral and somatic pain
17. Briefly describe the author's approach to the liabilities of the GOTO statement
18. Describe what happened at...?
19. Determine the author’s point of view
20. Determine the next number in a sequence
21. Discuss the nature of socialism.
22. Explain how Timothy saved Phillip's life.
23. Explain in one's own words how to create a query in a database.
24. Explain in your own words what a recessive gene is.
25. Explain in your own words what do you mean by the term economics?
26. Explain in your own words what is meant by mercantilism.
27. Explain the causes of historical events
28. Explain the communicative property.
29. Explain the double meaning the author attributes to the term "successive action descriptions"
30. Explain the possible uses described in class for the space to the left of the vertical line.
31. Explain the process (paraphrase) for finding the perimeter of a rectangular garden
32. Explain what a poem means.
33. Explain what the author means by the statement "our intellectual powers are geared to master static relations.
34. Express your opinion of 'Drugs in Sport' through poetry.
35. From a blueprint to describe the article depicted.
36. Given a graph of production trends in automobiles, describe what the graph represents in a memo to your boss.
37. Given these five corrosion inhibitors, V, W, X, Y, Z, identify which are passivators
38. Illustrate this caption: "Olympic as a Media Event in the Information Society"
39. Illustrate what you think the main idea was.
40. In one sentence explain the main idea of a written passage.
41. In one sentence illustrate the main point of a written passage.
42. Interpret the pictures.
43. Outline in your own words how the Leggo's Tomato Paste advertisement sells their product.
44. Outline the most important insight of the tale.
45. Outline the mechanisms employed by bacteria allowing them to evade phagocytic destruction.
46. Outline an "attack plan" that will allow you to test your hypothesis using computational molecular modeling tools
47. Paraphrase an important speech
48. Paraphrase what Hamlet is saying in his soliloquy.
49. Paraphrase the poem in your own words
50. Paraphrase this poem about Sheik Schubli, explaining what he was saying about the characteristics of a friend
51. Describe the output of this program fragment:
52. Classify types of corrosion inhibitors
53. would you classify the zener diode in this circuit as a series voltage regulator or a shunt voltage regulator
54. Classify the following protocols: IP, TCP, HTTP according to the ISO OSI classification
55. Classify the following substances as particle, atom, element, molecule, compound
56. How would you classify the geographic location of your school
57. Classify animals into two groups.
58. Classify celebrations into family and community categories.
59. Classify frogs toads and other amphibians.
60. Restate main idea of story in own words.
61. Restate the Olympic motto in your own words.
62. Retell the story in your words.
63. Rewrite the principles of test writing.
64. Rewrite you predicted equation for the ideal gas law
65. Rewrite the markov matrix to account for this slight change.
66. Rewrite the sentence in your head, following the direction given below
67. Rewrite a part in the poem using a different speaker
68. Select the best title for a passage
69. State in your own words the rule for balls and strikes in baseball.
70. State in your own words where in the library to find previous issues of journals that are no longer in the display racks.
71. Can you state in your own words what the problem is asking you to do
72. State in your own words what the author means when he says
73. Summarize a historical document
74. Summarize a story in own words.
75. Summarize Jem’s beliefs about Boo Radley
76. Summarize the basic tenets of collaborative conservation.
77. Summarize the basic tenets of deconstructionism.
78. Summarize this magazine article.
79. Tell how Phillip kept himself alive after Timothy died.
80. Tell in your own words how the setting of the story made it more interesting.
81. Tell in your own words the beginning of the book.
82. In your own words, tell what is meant by the definition of an isosceles triangle.
83. In your own words, how would you define transferable skills
84. Translate a written text aloud from L2 to English.
85. Translate an equation into a computer spreadsheet.
86. Translate an equation into a spreadsheet formula.
87. Translate the following passage from The Iliad into English.
88. Use the E-pH or Pourbaix diagram of Cr to determine the control current and potential for the anodic protection of a S43000 stainless steel vessel.
89. Compare and contrast two treatments of either drapery or animals by Greek sculptors, establishing their artistic contexts
90. Briefly compare this way of testing the satisfiability of to the more standard way using SLD Resolution, as in Prolog
91. How would you compare SAS with the Visual Basic Application and SPSS for the processing and analysis of mainframe data
92. Describe and compare the role of Islamic law in the legal systems of Malaysia and Pakistan
93. Express the rule in function notation
94. Express the following systems in matrix form
95. Express the following percentages as fractions in simplest form
96. Explain the whole method of crushing
97. In your own words, how would you summarize the story up
98. Summarize the main point of these two texts in your own words
99. Outline the aim of two studies within the
100. Outline your experience in working with service users

**Application**

1. Apply laws of statistics to evaluate the reliability of a written test.
2. Apply shading to produce depth in drawing.
3. Apply the rule of law to a new situation
4. Apply the storytelling technique here to a little story of your own.
5. Apply your understanding the Olympic spirit to develop a new motto or slogan.
6. Show the value of x after running this program fragment
7. Calculate for x in the following equation: 12x=36
8. Calculate the deflection of a beam under uniform loading.
9. Calculate the number of sacrificial anodes that would be required to form a calcareous deposit on a steel pile immersed in seawater.
10. Calculate the rate of habitat fragmentation within the Colorado Front Range in the last decade.
11. Can you apply the method used to some experience of your own . . .?
12. Can you group by characteristics such as . . .?
13. Categorise the pictures and add them to the wall display.
14. Choose a country that does not compete at the Olympics and explain why that country is not an Olympic member.
15. Choose any U.S. president and explain how he exercised his power as Commander in Chief of the Armed Forces.
16. Compute the area of actual circles.
17. Compute the volume of a cylinder
18. Construct a model to demonstrate how it will work.
19. Demonstrate how the seasons occur.
20. Demonstrate how this could work in an industry setting?
21. Demonstrate using the OPAC and your knowledge of library organization to find a book about turtles.
22. Demonstrate how your unit’s specific student learning outcomes are linked to the mission of UAM.
23. Derive a kinetic model from experimental data.
24. Describe an experiment to answer the question of the effects of weight on the fall of an object.
25. Construct a market strategy for your product using a known strategy as a model.
26. Construct a simple payroll program that computes net pay for those working 40 hours or less and net pay for those working over 40 hours.
27. Draw 3 pictures showing the beginning, middle and ending of the story.
28. Draw a picture of the bears' house.
29. Draw a picture of the gown Cinderella wore to the ball.
30. From the information given, can you develop a set of instructions about . . .?
31. How could someone modify the Cornell Method to make it effective for researching a topic for a paper?
32. How might lifestyles in the United States be modified if a more socialistic system were adopted?
33. If notes are available on the professor’s website, how should those notes be used?
34. In a teaching simulation with your peers role-playing 6th grade students, demonstrate the principle of reinforcement in classroom interactions and prepare a half page description of what happened during the simulation that validated the principle.
35. Make a diorama to illustrate an important event.
36. Make a paper-mache map to include relevant information about an event.
37. Make a scrapbook about the areas of study.
38. Make up a puzzle game using the ideas from the study area.
39. Model an Olympic Village for the new Millenium.
40. Participate in a mock election
41. Predict what happens to X if Y increases.
42. Pretend you are one of the characters in the book. Write a diary about the happenings in your life for two consecutive days.
43. Relate the principle of reinforcement to classroom interactions.
44. Relate topographic map to field features
45. Relate your most powerful story where your use of technology in the classroom has yielded a significant outcome with a student or students
46. How would you relate position concept with position strategy
47. Show, through role-play, the final scene in the novel.
48. Solve a math problem using familiar procedure or formula
49. Solve for the ten following fraction multiplication problems. Please make sure to show all your work.
50. Would you solve these two problems using similar approaches
51. Use Laplace to solve the following problems
52. Can you solve the following problem
53. How to specify the impact energy and impact test temperature of the material in the Code
54. Specify the thickness of a galvanized coating based on atmospheric data? (level 2)
55. Specify the thickness of a galvanized coating based on atmospheric data? (level 2)
56. How does one specify the syntactical restrictions
57. Specify a deterministic finite automaton (DFA) recognizing the
58. Take a collection of photographs to demonstrate a particular point.
59. Use a costing model to compute prices on goods to maximise profits and minimise costs.
60. Use a manual to calculate an employee's vacation time.
61. Use the distance travelled and cost of petrol to calculate the cost of a coach trip using a spreadsheet.
62. Use the rule for a semicolon in a sentence.
63. What factors would you change if . . .?
64. What questions would you ask if . . .?
65. Who can use what we know about sonnets and finish this poem?
66. Would this information be useful if you had a . . .?
67. Sketch a prediction of the field lines for the arrangement of electrodes shown in Figure 2
68. Sketch the blackbody curves for radiation emitted from the Sun and the Earth
69. Sketch a typical absorption process in a packed column with regeneration in form of
70. sketch a graph of velocity versus time for the upward and downward parts of a ball’s flight
71. How do teenagers use code in emails and instant messages?
72. Use the second-derivative test to determine whether critical points where f0(x) = 0 yield relative maxima or relative minima
73. Use the unification algorithm to determine whether the following two terms are uni_able. Show intermediate step
74. Use tableau methods to prove that the following tableau is valid.
75. Use a sketch too find the exact value of each expression
76. Use Gauss’ Law to find the electric field between the plates when the charge on them is Q
77. What happens if you restrict a determinental process to a subset R S?
78. What would happen if the rainfall doubled in your state next year
79. What happens if you change the direction of the displacement (but not its size)?
80. What would you do if a heavy growth of bacteria had occurred
81. What would result if …
82. What approach would you use to prove the truth of the below statement
83. Construct one Turing Machine for computing each of the following functions
84. Construct a Venn diagram to determine the validity of the given argument.
85. Construct a dictionary and a truth table to determine if the following statements are logically equivalent
86. How can we construct H given I, B and E?
87. Construct a family of networks to demonstrate that the number of different mincuts in a network can be exponential in the size of the network
88. How do you construct foliations on a given manifold M?
89. How to Construct the Optimal Maintenance Problem
90. How to construct simple solutions when problems are complex?
91. Construct the 4-Bit Ripple carry Adder Circuit
92. Construct an analog circuit with the following specifications
93. Construct the Data Path of the RISC Processor given in the previous question
94. How do we construct agents that can deliver. “good” assistance
95. How to construct the selected information system
96. Show how they have used creative writing strategies appropriate to the format
97. Show how to modify Fitch's algorithm given in class today to accommodate non-binary
98. Show how E-CRM can be used to improve marketing positioning as explained in the article
99. How would you show your understanding of the ABC process
100. Show me how you would check the brake lights are working on this car

**Analysis**

1. Analyze safe and dangerous aspects of these features.
2. Analyze the characteristics of frogs.
3. Analyze the movements and sounds of a frog.
4. Analyze the problem, identifying its variables.
5. Break down the components of a standard film camera and explain how they interact to make the machine work.
6. Break down the main actions of the story.
7. By comparing the map of the tectonic plates to the earthquake map, what inferences can you make?
8. Can you compare your . . . with that presented in . . .?
9. Can you distinguish between . . .?
10. Can you explain what must have happened when . . .?
11. Can you find four different feelings Pa Lia had during the story?
12. Compare and contrast animals that the class has made.
13. Compare and contrast our school to other communities.
14. Compare and contrast preview questions, clarifying questions and anticipated exam questions, including how each relates to the notetaking process?
15. Compare and contrast the waterfall model with the prototyping model.
16. Compare and contrast two characters in the book.
17. Compare fall and spring
18. Compare herbatious and carnivorous animals on a Venn diagram.
19. Compare how different children come to school.
20. Compare the place where the story happened with where you live.
21. Compare this book to the last book you read.
22. Compare three celebrations using a Venn diagram.
23. Compare two dog food commercials. What is the difference between them and how do they both sell their products?
24. Compare two of the characters in this book.
25. Conduct an investigation to produce information to support a view.
26. Contrast building in the coastal zone with building in a river floodplain.
27. Contrast Olympic athletes of today with athletes of past Olympic games.
28. Differentiate between .call by value and call by reference.
29. Differentiate printf function calls for displaying prompts and for echoing data.
30. Distinguish between micro and macro economics.
31. Distinguish facts from hypotheses
32. Divide the story into initiating event, rising action, climax, and falling action
33. Examine what helps to make a good Olympics? Think about money, schedules, sports and people.
34. Explain the term conjugal families, by making reference to the different types of societies to which they could belong.
35. analyze the positive and negative points presented concerning the abolition of guns and write a brief (2-3page) narrative of your analysis.
36. Differentiate the passages that attacked a political opponent personally, and those that attacked an opponent's political programs.
37. How does your pet differ from other animals?
38. How does...compare/contrast with...?
39. How is . . . similar to . . .?
40. How was life different in your town 100 years ago?
41. How was this similar to . . .?
42. How would this story be different if it had happened in a different country?
43. How would you distinguish between polymyositis and viral myositis in a 42-year-old man with weakness and a rash?
44. If your story happened in a foreign land, compare that land to the United States.
45. If your story occurred long ago, compare that time with today in a good paragraph. If it was a modern story, compare it with a long time ago and tell what would be different.
46. Investigate innovations that can enhance future Olympics.
47. Make a diagnosis or analyze a case study.
48. Select the athlete of the century and analyze why you chose this person.
49. If interest were compounded monthly instead of daily, what would the difference in interest be?
50. What are the parts or features of...?
51. Distinguish between the following giving suitable examples
52. Distinguish between abstract date types (ADTs) and implementations of ADTs
53. How do you distinguish between the binge-eating/purging type of anorexia nervosa and bulimia nervosa, purging type
54. How does Tdap differ from Td vaccine
55. How does autonomy differ with age
56. In what ways do female offenders differ from male offenders
57. in what ways trajectory patterns differ according to ABC
58. How do psychologists differ in their general attitudes toward third party presence
59. How did Chinese newspapers differ from US
60. How does public health reporting differ today from reporting two years before
61. How does an endorsement differ from a teaching certificate
62. How do we differentiate between genuine buyer and undercover buyer
63. How do we differentiate between an interview and an interrogation?
64. Differentiate between the following terms along with appropriate examples
65. Differentiate the following functions using the quotient rule
66. Can you differentiate between ethnic Chinese and ethnic Koreans
67. How would you differentiate obstructive apneas from central apneas
68. How would you differentiate a project from a program
69. How is scientific progress similar to Darwinian evolution according to Kuhn
70. in what ways is oxyperoxidase similar to other oxygenated heme proteins, and how does it differ
71. In what ways are foxes similar to wolves and dogs
72. How are the adolescents with ASD similar of different from each other in their sources of communication breakdowns ?
73. Examine the data in the EMPLOYEES and DEPARTMENTS tables
74. Examine the differences between the model excluding the most influential observation and the model including all observations.
75. Critically examine the strengths and weaknesses of the positivist paradigm
76. Examine differences between the means of each individual switching factor.
77. Examine carefully the given map extract on the following page to help you work out the exercise below.
78. Inspect each of these Boolean expressions, and determine whether each one is a sum of products, or a product of sums
79. Inspect the size of the loaded data: how many objects and variables does the iris data matrix contain?
80. Can you distinguish for me between what is reasonably achievable and what are exaggerations
81. Can you distinguish aged rums from unaged rums by smell
82. Can you distinguish pot still rums from column still rums by taste
83. Inspect the pattern of GPS positions relative to the “known” published value
84. How does your agency inspect against its expectation for operators
85. How to inspect and maintain fresh water plumbing systems properly
86. Can you analyze the fractures and determine which should be repaired surgically
87. Can you please analyze modern contemporary culture and tell me how your work plays a significant part in the remains of our post-modernist culture ?
88. How effectively did students analyze course content on race, class, and privilege through autobiographical reflection
89. How do you analyze a marginally RCT
90. Contrast the treatment of "well known" trademarks provided by Paris Article and by TRIPS Article
91. Contrast the Valles Marineris on Mars with the Grand Canyon
92. How does Hawaii contrast and compare to Florida?
93. Investigate whether or not the system can be solved for x; y; z
94. Investigate (pointwise) convergence and absolute convergence of the given series of functions
95. How would you investigate this patient
96. How would you investigate this abnormality
97. What can I do to investigate this problem further
98. how the investigation was undertaken
99. How would you differentiate between heterodox and mainstream
100. How do I differentiate between "deceive" and "cheat on"?

**Synthesis**

1. Apply and integrate several different strategies to solve a mathematical problem (not according to one formula(
2. Can you create new and unusual uses for . . .?
3. Can you design a . . . to . . .?
4. Can you develop a proposal which would . . .?
5. Can you invent another character for the story?
6. Can you see a possible solution to . . .?
7. Can you write a new recipe for a tasty dish?
8. Choose a character. Rewrite a scene from the story from this character's point of view.
9. Combine any two sports to develop a new Olympic sport.
10. Combine elements of drama, music, and dance into a stage presentation.
11. Compose a class story.
12. Compose a dialogue between Atticus Finch and Tom Robinson when they first met.
13. Compose a rhythm or put new words to a known melody.
14. Compose a simple rap or rhyme about zoo animals.
15. Compose music for a frog play.
16. Compose a complete C program that reads text strings from a text file into a suitable data
17. Compose a device that would assist an athlete in their training.
18. Compose an original work which incorporates five common materials in sculpture.
19. Compose your own note-taking design that incorporates all theadvantages of the Cornell Method.
20. Create a chart that compares things that use electricity and things that do NOT use electricity.
21. Create a new product. Give it a name and plan a marketing campaign.
22. Create a new song for the opening line of "Mary had a little lamb".
23. Create a set of guidelines to determine the points of a plant susceptible to localized corrosion.
24. Create a storyboard for a sequel to your book. Use the same characters.
25. Create an equation to represent the solution to this problem.
26. Create and perform a play about frogs.
27. Create plan of local environment by drawing around boxes.
28. Design a building according to given specifications
29. design a cost effective strategy to generate reliable data.
30. Design a machine to perform a specific task.
31. Design a new animal to live in the jungle.
32. Design a poster for this book.
33. Design a testing scenario to assess the susceptibility of an alloy to be used in a given environment?
34. Design and make an animal that moves.
35. Design costumes for the characters.
36. Design the architecture of the software system based on the requirements defined in the Software Requirement Specification document
37. Develop a hypothesis.
38. Develop a plan for a new Olympic Bid System.
39. Develop a SQA Plan for a software development project which is defined in the attached document.
40. Develop a way to teach the concept of "adjectives".
41. Develop one plausible ending for all three short stories below.
42. Devise a new economic system based on the ones that already exist.
43. Devise plans to market or make artwork more valuable.
44. Draw a painting that uses various principles of perspective to achieve its effect.
45. Explain how the biological concept of symbiotic relationships could be used to help solve socially created problems like water pollution, overflowing garbage landfills, or homelessness.
46. Explain why it is likely that a matriarchal family system would be found in a matrilocal or matrilineal society.
47. Following the identification of the forms of corrosion that were involved in a failure, recommend a solution to avoid this failure?
48. Given two opposing theories design an experiment to compare them.
49. How could we determine the number of pennies in a jar without counting them?
50. How could you re-write this story with a city setting?
51. how do the clauses and expressions given in the article which replace GOTO statements, lead to the three traditional control statements
52. How many ways can you . . .?
53. How would the U.S.A. be different if the South had won the Civil War?
54. How would you change the story to create a different ending?
55. How would you create/design a new...?
56. How would you restructure the school day to reflect children's developmental needs?
57. Identify one problem in the book and give an alternate solution one not given by the author.
58. If you had access to all resources how would you deal with . . .?
59. Integrate training from several sources to solve a problem.
60. Invent a machine to do a specific task.
61. Justify your choice of data structure.
62. Make a radio announcement that advertise the book. Write it out.
63. Make up a new language code and write material using it.
64. Name one character. Rewrite the story from this character's point of view.
65. Organize this book into three or more sections and give your own subtitle for each section.
66. Plan a research paper on a given historical topic
67. Predict the differences of a planet with no seasonal changes.
68. Prepare a book jacket that illustrates the kind of book as well as the story.
69. Pretend you are a librarian recommending this book to someone. Write a paragraph telling what you would say.
70. Revise and process to improve the outcome.
71. Revise how to complete a complex task in order to improve the outcome.
72. Revises and process to improve the outcome.
73. structure, sorts the list in ascending order, displays the list on the screen and stores the list
74. Suppose Phillip wasn't rescued shortly after Timothy's death. How long could he have survived?
75. Using information from the book about one of the main characters, rewrite the ending of the book.
76. What ideas can you add to...?
77. What might happen if you combined...?
78. What solutions would you suggest for...?
79. What would happen if . . .?
80. What would you predict/infer from...?
81. Why don’t you devise your own way to deal with . . .?
82. Why not compose a song about . . .?
83. Write a C program that accepts integer inputs from the screen, computes the total and average values; and displays the values on the screen.
84. Write a letter to the editor on a social issue of concern to you.
85. Write a logically organized argument in favor of a given position.
86. Write a poem about this book.
87. Write a set of rules to prevent what happened in the story.
88. Write a short story relating a personal experience in the style of a picaresque novel.
89. Write a song about 'Old MacDonald' who had a bulldozer instead of a farm.
90. Write an essay in not more than 250 words about India and Technological Advancement. Use active voice as much as possible.
91. How do you combine the results of various individual tests to determine whether a student can progress to a next semester / phase / year?
92. How can we combine and abstract facts about a software system to create new knowledge?
93. How to combine virtual symmetry and symbolic model checking effectively
94. How do we efficiently combine multiple subject graphs to construct an aig with choice
95. how do we devise a way to enforce the laws
96. can you devise an explicit formula for
97. Do you devise a systematic approach in order to get larger progressions
98. Develop and execute a program for this exercise
99. Develop a network based on the following information: Activity Immediate predecessors
100. How could we develop a coherent measure of success for organisations engaged in this type of activity with young people?

**Evaluation**

1. After designing an experiment, examining the results, and drawing conclusions, determines in what ways the experiment could be conducted more effectively in order to draw more productive conclusions in the future.
2. After examining the videotape of a play in a football game, determine the degree to which the defensive team performed effectively and
3. After solving a problem, determine the degree to which the problem was solved as efficiently as possible
4. Appraise data in support of a hypothesis.
5. Appraise the speech's effectiveness based upon the class' criteria.
6. Are there students who should not use the Cornell method?
7. Are you a . . . person?
8. Assess the appropriateness of an author's conclusions based on the evidence given.
9. Assess the importance of understanding seasonal patters.
10. Assess the relative effectiveness of different graphical representations of the same data or biological concept.
11. Assess the strengths and weaknesses of the current Olympics and recommend action that should be taken in future Olympics: What can be
12. Award the contract to the best proposal. Rank the principles of "good sportsmanship" in order of importance to you.
13. Can you defend the idea that Simon's incident with the pig's head is the most mystical in the story?
14. Can you defend your position about . . .?
15. Choose a paint schedule for the maintenance of an outdoor structure? (level 2)
16. Choose and illustrate the two most important events in the story.
17. Conclude and support which economic system leads to a higher standard of living.
18. Construct a poster that will advertise your new food product in an exciting and irresistible way.
19. Critique an experimental design or a research proposal.
20. Critique the other student's (or your own) speech, based on the criteria we have studied this semester.
21. Decide whether you are in favor of building on a floodplain; defend your position in a debate.
22. Decide whether you could have survived on the island blind and alone. Write about things that would have been challenging making sure to
23. Decide whether you learned enough about electricity from this book.
24. Decide which candidate would best fill the position of principal.
25. Decide which course of action was most effective
26. Decide which method of solving a problem was most effective
27. Defend the following claim: The Cornell method works so well that it could turn even a poor lecture into a valuable learning experience.
28. Defend your use of a specific strategy in solving the problem.
29. Describe the economic consequence of a neolocal society. Support your description with information you have learned from this course.
30. Design a healthy menu that you think most people would enjoy using the healthy eating guide.
31. Determine if a character’s actions were heroic
32. Do you agree...?
33. Do you think . . . is a good or a bad thing?
34. Establish criteria for making this choice and defend your final selection.
35. Evaluate a work of art, giving the reasons for your evaluation.
36. Evaluate appropriate and inappropriate actions of characters.
37. Evaluate board games and justify why rules are important.
38. Evaluate the corrosion monitoring needs of a chemical processing plant
39. Evaluate the level of galvanic coupling between two metals using basic kinetic information? (level 4)
40. Evaluate two Internet sources of information about the Egyptians. Which would be a better choice for your purpose and why?
41. Evaluate whether their model is a true representation of the local environment.
42. Evaluate your own or a peer's essay in terms of the principles of composition discussed during the semester.
43. Examine the stated positions of both major political candidates with regard to a particular issue and state good reasons (based on principles discussed in class) for why one candidates position is more likely to be effective than the other's.
44. Explain and justify a new budget.
45. Explain choices made in making recommendations to an end user.
46. Given an argument on any position, enumerate the logical fallacies in that argument.
47. Given the data available on a research question, take a position and defend it.
48. Given the data we've looked at on this topic, evaluate how appropriate this conclusion is and defend your answer.
49. Given the two solutions to the stated programming problem, rate the solutions in terms of efficiency and readability.
50. Given three possible approaches to implement the defined system, discuss the possible advantages and disadvantages of each approach.
51. Given two possible solutions, A and B, to solving the given software development problem, decide on the best solution. Give your justification.
52. How effective are . . .?
53. How would you decide about...?
54. How would you feel if . . .?
55. How would you have handled . . .?
56. improved, reformed or rejected?
57. In a given clinical situation, select the most reasonable intervention and predict the main effects and possible side effects.
58. Is there a better solution to . . .?
59. Judge aesthetic qualities and relationship to future values.
60. judge the difficulty level for yourself.
61. Judge the value of . . .?
62. Judge whether it would be possible to survive on an island alone and blinded. Write about it.
63. Judge whether Olympic Ideals are realistic or unrealistic for the contemporary elite athlete.
64. Justify and nominate ways to prevent animal extinction.
65. Justify the title To Kill a Mockingbird .
66. Listen to two classmates conversing on tape and critique their performance on the basis of the skills covered this semester.
67. Place the following in order of priority...
68. Predict what will happen next in.
69. Predict whether Phillip will ever go back to visit the Cay after several years of recovery. Assuming he gets his sight back.
70. Recommend how our classroom or playground could be improved.
71. Select the best proposal for a proposed water treatment plant.
72. Select the most effective solution.
73. suggest ways in which it could have responded more effectively
74. decide about the most exciting part of the book. being sure to give at least three reasons why?
75. Use a judge and jury to discuss the statement 'Children enjoy Anthony Browne's books because of the illustrations'.
76. Using straight value depreciation, decide between two copper-nickel alloys for the design of a heat exchanger.
77. Using the basic principles of socialism discussed in this course, evaluate the US economic system by providing key arguments to support your judgment.
78. Was Hemingway a great American writer? First you will need to define greatness.
79. What changes to . . . would you recommend?
80. What criteria would you use to assess the validity of a business contract?
81. What criteria would you use to assess...?
82. What do you think about . . .?
83. What is part of this book did you like best. Tell why you like it?
84. What is the most important...?
85. Which of the following actions would probably be least effective in determining the answer?
86. Which of the two algorithms, bubblesort or quicksort, is more efficient? Justify your answer.
87. Would you have liked to have had Cinderella for a sister? Explain why or why not.
88. Write a list of criteria to judge the Willy raps.
89. Revise the story and specify the type of audience that would enjoy this book.
90. Estimate the epicentral coordinates of the earth-quake
91. Estimate the risk of serious illness for consumers in different susceptible population groups
92. Estimate expenditure on HIV/AIDS education
93. Estimate the difficulty of the question
94. Estimate roughly how long the collision process takes
95. What do you think is the best thing about coming to Viestal?
96. What do you think is the mass and why
97. What do you think are the strengths of using integrated math in your program?
98. Do you think that this statement conveys the idea that i am trying to portray
99. How do you think the community should grow or change
100. How Do You Think One Should Reason in Making Ethical Decisions in Business
